# When accurate prediction models yield harmful self-fulfilling prophecies

**DOI:** 10.1016/j.patter.2025.101229

**Published:** 2025-04-11

**Authors:** Wouter A.C. van Amsterdam, Nan van Geloven, Jesse H. Krijthe, Rajesh Ranganath, Giovanni Cinà

**Affiliations:** 1 Department of Data Science and Biostatistics, Julius Center for Health Sciences and Primary Care, University Medical Center Utrecht, University of Utrecht, Heidelberglaan 100, 3584 CX Utrecht, the Netherlands; 2 Department of Biomedical Data Sciences, Leiden University Medical Center, Albinusdreef 2, 2333 ZA Leiden, the Netherlands; 3 Pattern Recognition & Bioinformatics, Delft University of Technology, Mekelweg 5, 2628 CD Delft, the Netherlands; 4 Courant Institute of Mathematical Science, Department of Computer Science, Center for Data Science, New York University, 251 Mercer St., New York, NY 10012, USA; 5 Department of Medical Informatics, Amsterdam University Medical Center, Meibergdreef 9, 1105 AZ Amsterdam, the Netherlands; 6 Institute for Logic, Language and Computation, University of Amsterdam, Amsterdam, the Netherlands; 7 Pacmed, Amsterdam, the Netherlands

**Keywords:** prognosis, deployment, monitoring, decision support techniques, causal inference, data drift

## Abstract

Prediction models are popular in medical research and practice. Many expect that by predicting patient-specific outcomes, these models have the potential to inform treatment decisions, and they are frequently lauded as instruments for personalized, data-driven healthcare. We show, however, that using prediction models for decision-making can lead to harm, even when the predictions exhibit good discrimination after deployment. These models are harmful self-fulfilling prophecies: their deployment harms a group of patients, but the worse outcome of these patients does not diminish the discrimination of the model. Our main result is a formal characterization of a set of such prediction models. Next, we show that models that are well calibrated before and after deployment are useless for decision-making, as they make no change in the data distribution. These results call for a reconsideration of standard practices for validation and deployment of prediction models that are used in medical decisions.

## Highlights

• Some prediction models yield harmful self-fulfilling prophecies when used for decisions
• These models harm patients but display good discrimination post-deployment
• Models that retain calibration post-deployment may be useless for decision-making
• The evaluation of prediction models for decision support must align with their final aim

## The bigger picture

To tailor treatment decisions to individual patients, many researchers develop prediction models. These models assess a patient’s risk of an adverse outcome, such as a heart attack, based on their characteristics. Many believe that the best prediction models for decision-making are those that have the highest predictive performance, e.g., discrimination—the ability to assign higher risks to patients with the outcome compared to those without. Common advice is to keep evaluating discrimination after the model’s implementation to ensure effective decision-making. We show, through a clinical example and mathematical proofs, that this belief is flawed because of the existence of so-called “harmful self-fulfilling prophecies”: prediction models that retain good discrimination after implementation and yet harm patients when used for decision-making. The takeaway is that rather than relying on discrimination, we should assess models based on their impact on treatment decisions and patient outcomes.

## Teaser

Prediction models are often developed with the aim to guide treatment decisions, but can they cause harm? This study reveals that even models with good post-deployment performance—such as strong discrimination or calibration—can negatively impact patient outcomes. By identifying harmful self-fulfilling prophecies, the authors argue that evaluating prediction models should focus on their impact on treatment decisions and patient outcomes, not just predictive accuracy, to ensure they improve decision-making and patient care.

## Introduction

Clinicians and medical researchers frequently employ outcome prediction models (OPMs): statistical models that predict a certain health outcome based on a patient’s characteristics.^1^ Researchers develop OPMs to provide information to clinicians so they may use this information in difficult treatment decisions (e.g., Salazar et al.^2^). In some cases, clinicians will treat patients with a bad expected outcome more aggressively, for example, by giving cholesterol-lowering medication to patients with a high predicted risk of a heart attack.^3^^,^^4^ In other cases, for instance, when the treatment is burdensome or scarcely available (e.g., ventilator machines in the intensive care unit during a pandemic), clinicians may reserve treatment for patients with a good predicted outcome.

Many such OPMs are added to the protocol of care by designing specific thresholds for specific actions.^3^ If the predicted outcome is above or below the threshold, a certain action is taken, e.g., the patient receives a more aggressive therapy. The basis for including an OPM in a care protocol is generally predictive accuracy in validation studies.^5^ In these validation studies, the OPM may or may not have been used to inform treatment decisions. While the difference between a clinical trial of an OPM’s deployment and the validation of performance metrics is appreciated in the medical literature, there are still notable examples where the latter is perceived to be sufficient to justify the implementation of OPMs in the protocol of care. This is reflected in several guidelines and reviews.^5^^,^^6^ Commonly used performance metrics are measures for discrimination and calibration, the latter being assessed much less frequently than the former.^7^

At first, it may seem that using OPMs for decision support is beneficial since giving more information should lead to better treatment decisions. However, implementing a prediction model for treatment decisions is an intervention that changes treatment decisions and, thus, patient outcomes. Whether this change in treatment policy improves patient outcomes is not determined by prediction accuracy in a validation study.^8^ For instance, in cases where a certain patient subpopulation historically received suboptimal care, an accurate OPM will predict a worse outcome for these patients compared to similar patients from a different subpopulation. If clinicians decide to withhold effective treatments (e.g., due to scarcity or perceived futility) to this underserved subpopulation based on the OPM’s prediction of a bad outcome, then the implementation of the OPM perpetuates biases and causes harm to these patients despite its accuracy. Moreover, the implementation of this harmful new policy brought about the scenario predicted by the OPM, as in a self-fulfilling prophecy. One concrete example where clinicians treat patients with a bad expected outcome less aggressively is in small cell lung cancer. Prognostic scores for patients with small cell lung cancer, such as the Manchester score,^9^ are specifically intended to not overtreat patients with a bad predicted outcome because this is expected to be futile.^10^^,^^11^

Recognizing that prediction model performance may change over time, across healthcare settings, and in certain patient subgroups, many call for increased monitoring of AI models, with model updating mentioned as the best approach.^12^^,^^13^^,^^14^ However, these approaches fall short, as they put the wrong metric upfront: prediction accuracy. We show that the value of a prediction model is not directly derived from its accuracy, and in some cases, having worse prediction accuracy over time is exactly what we want from a patient outcomes perspective. Focusing only on predictive performance might lead to the employment of a new policy that is harmful to patients or to the unduly withdrawal of a policy that was, in fact, beneficial.

In this article, we address the following questions. (1) Under what conditions is a new policy based on an OPM going to be harmful, meaning that it leads to worse outcomes than before using the model? (2) In what circumstances would such a harmful policy go undetected by measures of discrimination or calibration? In what follows, we provide a formalization of the case where patients with a high predicted probability of the outcome get treatment, where the outcome may be preferable (e.g., 1-year survival) or undesirable (e.g., a heart attack). Specifically, we examine the setting where a new OPM is supposed to “personalize” an existing treatment policy by considering additional features. The methods section provides a motivating example, notation, and definitions, and the results section presents the main results concerning OPMs that are harmful and self-fulfilling prophecies. We first show that even in a simple setup with a binary covariate, a non-trivial subset of OPMs yields harmful self-fulfilling prophecies. This means that such models cause harm but exhibit good discrimination on post-deployment data, meaning that naively interpreting this as a successful deployment leads to harmful policies. These theoretical results are paired with numerical experiments demonstrating that harmful self-fulfilling prophecies can occur without assuming extreme treatment effects or treatment effect heterogeneity. Next, perhaps surprisingly, we show that when an OPM is well calibrated on both (1) the historical data and (2) a validation study where the model is used for treatment decisions, the OPM is not useful for decision-making. Finally, after highlighting the shortcomings of validating and implementing OPMs based on predictive performance, we mention approaches to model building and validation that explicitly account for the *causal* effects of treatments on the predicted outcomes, avoiding such shortcomings.

Based on our results, several common practices in building and deploying OPMs intended for decision-making need revision. (1) Developing OPMs on observational data without regard for the historical treatment policy is potentially dangerous because the change in treatment policy between pre- and post-deployment is what determines the effect of the model on patient outcomes. (2) Implementing a personalized OPM is not always beneficial, even if the model is very accurate. (3) When monitoring discrimination prospectively after deployment, sometimes good discrimination means a harmful new policy and sometimes a beneficial one.

## Methods

### Motivating example of a harmful self-fulfilling prophecy

We start with a hypothetical example based on realistic medical assumptions that would result in an OPM yielding a policy that is both harmful, meaning patient outcomes are worse compared to before deployment, and self-fulfilling, meaning the OPM has good discrimination post-deployment. In Note S1, we provide a formal version of this example with corresponding equations and proof.

Consider the problem of selecting a subset of patients with end-stage cancer for palliative radiotherapy. Such treatment has side effects, and thus, domain experts advise reducing overtreatment in this population. To comply with this advice, a medical center needs to decide which patients will not be eligible anymore for the therapy. The medical center decides to give the therapy to patients with the longest expected overall survival, under the assumption that for these patients, the side effects are justifiable. To support this policy, researchers build an OPM to predict the probability of 6-month overall survival based on the pre-treatment tumor growth rate using historical patient records from the medical center. Fast-growing tumors are more aggressive, so these patients have a shorter survival overall. The medical center decides to implement this model to allocate radiotherapy and tests the model’s discrimination post-deployment.

The new treatment policy with the OPM is, thus, “treat patients with slow-growing tumors but not those with fast-growing tumors.” However, fast-growing tumors respond better to radiotherapy than slow-growing tumors,^15^ so the new OPM-based policy treats exactly the wrong patients: those who do not benefit from treatment still receive it, and those who would benefit from treatment do not, so deployment of the model is harmful. The contrast in survival between patients with fast-growing tumors and slow-growing tumors is only more pronounced post-deployment, meaning that, paradoxically, the OPM has good discrimination before and after deployment.

This potential for deploying harmful self-fulfilling prophecies by only relying on measures of predictive discrimination is clearly undesirable. We now provide a formal description of when these situations occur, revealing also a dual case where OPMs that provide benefits to patient subgroups show worse post-deployment discrimination.

### Notation and definitions

We assume a binary treatment T, a binary outcome Y, and a binary feature X∈X={0,1}. We denote the outcome obtained with setting treatment T to t as $Y_{t}$. An OPM is a function trained on historical data to predict the probability of the outcome of interest. We use $\pi_{i}\left(X\right)$ to denote a policy for assigning treatment, possibly conditional on X, with an index i to indicate what policy we are referring to. Throughout the paper, $\pi_{0}$ will be used to indicate the historic treatment policy that was in place in the data from which the OPM was developed.

We assume the historical policy is constant and deterministic, meaning that it is always equal to 0 or 1 (i.e., patients were always treated or never treated). Next we define what it means to craft a policy based on an existing OPM. We will be concerned only with *threshold-based policies*, namely policies that assign treatment based on a threshold λ∈R. In our setup, policies assign treatment to patients only if the expected outcome is above λ, which could mean either a desirable (e.g., 1-year survival) or undesirable (e.g., a heart attack) outcome.

Definition 1 (policy informed by OPM): let f:X→[0,1] be an OPM and λ∈R a threshold. We call $\pi_{f}$ a policy informed by f and define it as follows:

(Equation 1) $$\pi_{f}\left(x\right)=\left\{ \begin{array}{l} 1\;f\left(x\right)>\lambda \\ 0\;f\left(x\right)\leq \lambda \end{array}\right.\text{.}$$

Such policies describe the post-deployment scenario, when the OPM influences treatment assignment. This deployment will change some of the outcome distributions compared to pre-deployment. We distinguish probabilities pre- and post-implementation using subscripts: $p_{i}\left(.\right)$ with i=0 for the pre-implementation probabilities and i=f for the post-implementation probabilities. We now present the first key idea of this paper, namely the special class of OPMs whose predictions are reinforced upon implementation. We consider as a metric of discrimination the popular area under the receiver operating characteristic curve^16^ (AUC).

Definition 2 (self-fulfilling OPM): let f:X→[0,1] be an OPM and λ∈R a threshold, and let $\pi_{f}$ be the policy informed by *f*. Let $AUC\left(\pi_{i}\right)$ denote the AUC of this OPM on data generated with the historic policy $\left(\pi_{0}\right)$ or with the policy defined by $\left(\pi_{f}\right)$. We call the pair (f,λ) self-fulfilling if the AUC remains the same or increases post-deployment, namely iff

(Equation 2) $$AUC\left(\pi_{f}\right)\geq AUC\left(\pi_{0}\right)\text{.}$$

Finally, we specify what we mean with an OPM being harmful in comparison with the status quo.

Definition 3 (harmful OPM): let f:X→[0,1] be an OPM and λ∈R a threshold, let $\pi_{0}$ denote the historic treatment policy, and let $\pi_{f}$ be the policy informed by *f.*

We write the expected outcomes under the different policies as

(Equation 3) $$p_{i}\left(Y=1|X\right)=E_{T∼\pi_{i}\left(X\right)}p\left(Y_{T}=1|X\right)\text{,}$$

where i=0 denotes the historical distribution and i=f the distribution under $\pi_{f}$. We call f harmful for the group with X=x with p(X=x)>0 if the expected outcome of this group is worse under the new policy compared to the old policy, namely when Y=1 is preferable iff

(Equation 4) $$p_{f}\left(Y=1|X=x\right)<p_{0}\left(Y=1|X=x\right)$$

or when Y=0 is preferable iff

(Equation 5) $$p_{f}\left(Y=1|X=x\right)>p_{0}\left(Y=1|X=x\right)\text{.}$$

Note that this definition is for when deploying an OPM is harmful to a subgroup of patients, which, in general, is different from being harmful marginally, i.e., applying $\pi_{f}$ leads to worse outcomes on average. However, we will later see that in our setup with binary X, one of the two groups has the same outcomes pre- and post-deployment, so an OPM that is harmful to a subgroup will also be marginally harmful. When a policy informed by an OPM is both harmful and self-fulfilling, we have a worst-case scenario where the new policy is causing harm to a subgroup, but this, perhaps counterintuitively, does not result in a decrease in AUC post-deployment.

## Results

We now move to the main results, whose proofs can be found in Note S2.

The setting where a new OPM is supposed to personalize an already existing treatment policy by considering more features is encoded as follows: the new OPM considers a feature X that was previously ignored by the historical policy, specifically, $\pi_{0}$ is constant and deterministic. In addition, the new policy $\pi_{f}$ is not constant but varies with *X*.

### Harmful models may have good discrimination post-deployment

We state our main observation as an informal theorem.

Theorem 4 (informal main result): let $\pi_{f}$ be the policy informed by the OPM f using a threshold λ. Assume that (1) the historical policy $\pi_{0}$ is constant and deterministic, (2) the new policy $\pi_{f}$ is not constant, i.e., not always equal to 1 or 0, and (3) the marginal distribution of X is the same pre- and post-deployment: $p_{i}\left(X\right)=p\left(X\right)$ for i∈{0,f}.

Under these assumptions, a non-trivial subset of OPMs will demonstrate good post-deployment discrimination because they yield self-fulfilling prophecies and, at the same time, their deployment harms patients.

We proceed to characterize the contours of the subset of self-fulfilling and harmful OPMs.

Proposition 5 (self-fulfilling): suppose that the assumptions of theorem 4 hold. Furthermore, assume that the joint probabilities of X and Y are non-deterministic both pre- and post-deployment:

(Equation 6) $$0<p_{i}\left(Y=1,X=x\right)<1,\forall x\in X\text{.}$$

Then, the following two statements are true: (1) if the treatment effect is always positive, namely $\forall x\in X:p\left(Y_{1}=1|X=x\right)\geq p\left(Y_{0}=1|X=x\right)$, then (f,λ) is self-fulfilling, and (2) if the treatment effect is always negative, meaning $\forall x\in X:p\left(Y_{1}=1|X=x\right)<p\left(Y_{0}=1|X=x\right)$, then (f,λ) is not self-fulfilling.

Proposition 5 gives sufficient conditions for an OPM to yield a self-fulfilling prophecy. When Y=1 is preferable, meaning the new policy treats only those with a favorable predicted outcome (e.g., under resource scarcity), the sufficient condition is that the treatment effect is beneficial for all values of *X*. When instead Y=0 is preferable, meaning the “treat high-risk patients” setting, the sufficient condition is that treatment is detrimental for all values of *X*. Treatments that are detrimental for all values of X are less likely to be used in practice, as, most often, treatments are approved for use after they are proven to be beneficial on average with a randomized controlled trial (RCT). In this case of “treat high-risk patients,” self-fulfilling prophecies may still occur when the treatment is detrimental to a subgroup of patients. The assumption in proposition 5 that a treatment is always beneficial (or harmful) may hold in many cases. Typically, the *size* of the effect may vary over individuals, but that does not mean that a treatment is beneficial for patients with some values of X but harmful for patients with other values. For example, patients with a high risk of cardiovascular disease are expected to benefit more from preventative treatments, such as cholesterol-lowering medication, than patients with a low risk of cardiovascular disease, but a beneficial effect is expected in all patients. If the assumption does not hold, meaning that the treatment is beneficial for some patients and detrimental for others, then self-fulfilling prophecies may still occur, as shown later in the numerical experiments, but we can no longer provide sufficient conditions for when a self-fulfilling prophecy will occur for sure.

Remark 6: proposition 5 does not depend on the OPM’s discrimination in the historical data, meaning that models with “good” discrimination (i.e., high AUC) and “bad” discrimination (low AUC) are equally susceptible to yielding self-fulfilling prophecies under the conditions of the proposition.

Now we know when OPMs are self-fulfilling and thus have good post-deployment discrimination, but can these self-fulfilling OPMs also be harmful? Proposition 7 indicates that they can.

Proposition 7 (harmful): under the assumptions of theorem 4, when Y=1 is preferable, f is harmful for the group with X=x iff

(1) $\pi_{0}\left(x\right)=1$ and $\pi_{f}\left(x\right)=0$ and $p\left(Y_{1}=1|X=x\right)>p\left(Y_{0}=1|X=x\right)$ or
(2) $\pi_{0}\left(x\right)=0$ and $\pi_{f}\left(x\right)=1$ and $p\left(Y_{1}=1|X=x\right)<p\left(Y_{0}=1|X=x\right)$.

When Y=0 is preferable, the inequality signs reverse.

The conditions of this proposition indicate that, as one would expect, removing the treatment from the group with X=x is harmful iff $p\left(Y_{1}=1|X=x\right)>p\left(Y_{0}=1|X=x\right)$ (assuming Y=1 is preferable), i.e., if the effect of the treatment was positive for this group. Conversely, adding treatment to this group is damaging iff $p\left(Y_{1}=1|X=x\right)<p\left(Y_{0}=1|X=x\right)$ (when Y=1 is preferable), meaning that the treatment decreases the outcome for the group.

Remark 8 (harmful OPMs are marginally harmful): under the assumptions of theorem 4, OPMs that are harmful for one subgroup are also harmful on average, as the other subgroup’s treatment policy and outcomes do not change.

Taking proposition 5 on when OPMs yield self-fulfilling prophecies and proposition 7 on when OPM deployment is harmful together, we reach the perhaps surprising conclusion of theorem 4: even in the simple setup of binary treatment and binary *X*, some OPMs are both self-fulfilling prophecies, thus demonstrating good post-deployment discrimination, and harm a patient subgroup when deployed. We presented an example above, which we formalize in Note S1. In Table 1, we list the cases in which OPM deployment is harmful based on three pieces of information that are available post-deployment: (1) is Y=1 preferable or undesirable? (2) Was the historical policy “treat everyone” or “treat no one”? (3) Did the AUC of the OPM increase post-deployment compared to the AUC pre-deployment (i.e., is the OPM self-fulfilling)? Finally, we note that the performance of the OPM on the historical data does not feature in the assumptions or statement of proposition 7. This entails, contrary to common expectations, that a high performance on historical data, including external validation, provides no guarantee of whether the OPM-driven policy will be beneficial.

**Table 1:** Overview of when OPM deployment was harmful based on three pieces of information that were available post-deployment

Interpretation of Y=1 (and policy)	$\pi_{0}$	$\text{AUC}\left(\pi_{f}\right)-\text{AUC}\left(\pi_{0}\right)$	OPM deployment was
Desirable (treat low risk patients)	1 (treat everyone)	>0 (self-fulfilling)	harmful
	1 (treat everyone)	<0 (not self-fulfilling)	beneficial
	0 (treat no one)	>0 (self-fulfilling)	beneficial
	0 (treat no one)	<0 (not self-fulfilling)	harmful
Undesirable (treat high-risk patients)	1 (treat everyone)	>0 (self-fulfilling)	beneficial
	1 (treat everyone)	<0 (not self-fulfilling)	harmful
	0 (treat no one)	>0 (self-fulfilling)	harmful
	0 (treat no one)	<0 (not self-fulfilling)	beneficial

These results apply under the assumptions of theorem 4: the historical policy $\pi_{0}$ is constant and deterministic, the new policy is not constant, and the marginal distribution of X (a binary variable) is the same pre- and post-deployment. As an example, the top row corresponds to the motivating example in the introduction. Before deployment, the historical policy was to treat everyone; the new policy is to treat only patients with a favorable expected outcome. Post-deployment, the OPM has better discrimination than before deployment. Because the treatment decisions (and thus outcomes) for the patients with a good prognosis have not changed, an increased AUC post-deployment can only occur when the patients with a bad prognosis have even worse outcomes than before. Thus, the deployment must have been harmful. $\pi_{0}$, historical treatment policy (either treat everyone or treat no one); $\text{AUC}\left(\pi_{f}\right)$, AUC post-deployment; $\text{AUC}\left(\pi_{0}\right)$, AUC pre-deployment; OPM, outcome prediction model.

In examining such results, one may wonder what would be the size of the differences in AUC for the described harmful self-fulfilling OPMs in realistic data settings and if such OPMs may also occur if the assumption of the treatment being beneficial (or harmful) for all values of X does not hold. To answer these questions, we conducted a numerical experiment via the following data distributions:

(Equation 7) $$x∼B\left(p_{x}\right)\text{,}$$

(Equation 8) t∈{0,1},

(Equation 9) $$\eta =\beta_{0}+\beta_{x}x+\beta_{t}t+\beta_{xt}xt,\text{and}$$

(Equation 10) $$y∼B\left(\sigma \left(\eta \right)\right),$$

where $p_{x}$ is the proportion of data points with a positive attribute X, B the Bernoulli distribution, t is the historical treatment policy (which is always 0 or 1 according to our assumptions), σ is the sigmoid (logistic) function, and the β parameters encode the effects of x and t on the outcome. Setting these parameters, enforcing the assumptions of the theorem, and deciding whether a higher Y is better (e.g., 1-year survival) or worse (e.g., a heart attack) gives enough information to describe the pre- and post-deployment scenarios. Note that a non-constant policy $\pi_{f}$ entails that different treatments are now prescribed for the two groups defined by *X*; thus, further assumptions on the model and threshold λ are not needed. This allows us to calculate discrimination statistics pre- and post-deployment and to determine whether the new policy is harmful. By repeating the experiment for several values of the parameters—within reasonable ranges—one can investigate when harmful self-fulfilling policies arise.

**Figure 1:**
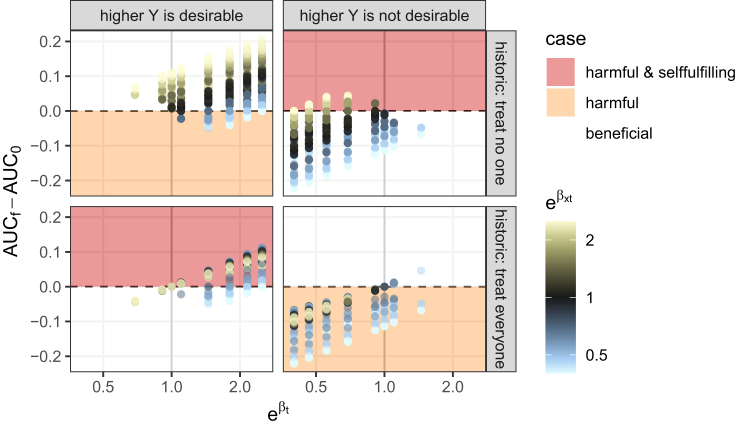
Results of the numerical experiment The treatment effect is reported on the horizontal axis on the odds ratio scale $\left(e^{\beta_{t}}\right)$, while the difference in AUC pre- and post-deployment is given on the vertical axis. When the said difference is positive, we have a self-fulfilling policy. An increase in AUC occurs when the difference in outcomes between the two groups increases post-deployment. Since our setup implies that only one group changes, this means that either the good-prognosis group got even better (a beneficial policy) or the bad-prognosis group got even worse (a harmful policy). The historic policy determines which group has a change in treatment policy and outcome. The four graphs reflect the different combinations of historical policy and outcome interpretation. Areas with (harmful) self-fulfilling prophecies are color coded. Each of the 8 areas corresponds to a row in Table 1. The points are color coded with the value of treatment effect interaction, again with an odds ratio $\left(e^{\beta_{tx}}\right)$.

The results match the theoretical findings and, furthermore, display that harmful self-fulfilling policies do occur in “common” circumstances without extreme treatment effects or extreme treatment effect interactions, as well as when the treatment effect is not of a constant sign. Figure 1 shows several instances of the experiment. A positive difference in AUC denotes a self-fulfilling policy, while harmful policies fall within a colored area. Inspection of the figure reveals several scenarios to be harmful and self-fulfilling in the top right and bottom left graphs. These scenarios can occur at different values of treatment effect (parameter $\beta_{t}$) and can even lead to an increase of AUC of >0.1. For Figure 1, we only kept settings where the treatment effect is beneficial on average. This removes several cases of harmful self-fulfilling prophecies but is more realistic, as treatments are generally only allowed on the market if their average effectiveness is demonstrated in RCTs. In Figure S3 in Note S3, all settings are presented. Furthermore, Figure S6 in Note S3 gives another visualization of the same experimental results, this time highlighting that harmful self-fulfilling prophecies occur in the absence of strong treatment effect interactions (i.e., treatment effect heterogeneity or the parameter $\beta_{xt}$).

Full details on the setup of the numerical experiment and further results can be found in Note S3, and the code to reproduce the results is available online (see data and code availability).

Note that the table and figures highlight a dual problem of harmful self-fulfilling, which we could call “beneficial self-defeating”: the case where AUC decreases post-deployment but the new policy is, in fact, beneficial. In this case, an overreliance on performance metrics might lead to another ill-advised decision: the withdrawal of a policy that was, in fact, beneficial.

### OPMs that are calibrated pre- and post-deployment are not useful for treatment decisions

Monitoring discrimination post-deployment and naively interpreting good post-deployment discrimination as a safe deployment is not a good strategy, as self-fulfilling prophecies have good post-deployment discrimination but can still be harmful depending on the context. Conversely, beneficial policies may have decreased post-deployment discrimination due to the desirable effect of improving patient outcomes.

We now turn to another key metric of OPMs predicting the risk of an outcome, calibration,^17^^,^^18^^,^^19^ and investigate how post-deployment calibration relates to harmful policies.

We use the following definition of calibration.

Definition 9: let p(X,Y) be a joint distribution over feature X and binary outcome Y and f:X→[0,1] an OPM. f is calibrated with respect to p(X,Y) if, for all α∈[0,1] in the range of *f*, $E_{X,Y∼p\left(X,Y\right)}\left[Y|f\left(X\right)=\alpha \right]=\alpha$.

The OPM can be calibrated on the pre-deployment historical distribution and/or the post-deployment distribution. Theorem 4 states that harmful OPMs can have good pre- and post-deployment discrimination, but can they also have good calibration?

The following theorem shows that OPMs that are calibrated pre- and post-deployment do not lead to better treatment decisions.

Theorem 10: let f be an OPM that is calibrated on historical data and $\pi_{f}$ be non-constant. Such an OPM is calibrated on the deployment distribution iff, for every x∈X,

(Equation 11) $$\pi_{0}\left(x\right)=\pi_{f}\left(x\right)\;\text{or}\;p\left(Y_{1}=1|X=x\right)=p\left(Y_{0}=1|X=x\right)\text{.}$$

Note that this entails that for all x∈X, either the treatment policy does not change or it changes where it is irrelevant because, for that value of X, the treatment effect is zero. Both cases imply that the implementation of the OPM is inconsequential. This may seem counterintuitive, but an OPM being calibrated both before and after deployment means the distribution has not changed, so the policy remains the same, or the policy was changed where it is irrelevant (i.e., no treatment effect). Therefore, an OPM that is calibrated on the development cohort that remains calibrated post-deployment is not a useful OPM.

### Related work

The intuition that deploying models for decision support is an intervention that requires causal evaluation methods goes back to at least the 90s,^20^ and previous work noted that prediction accuracy does not equal value for treatment decision-making.^8^^,^^21^^,^^22^ Here, we take the additional step of exactly characterizing the set of prediction models that yield harmful self-fulfilling prophecies. The idea that model deployment changes the distribution and affects model performance was noted in several lines of previous work. Several authors noted that model performance may degrade over time due to the effect of deployment of the model,^23^^,^^24^ but we study the case where model performance does *not* degrade but the implementation of it still causes harm. Also, we find that degraded discrimination may indicate a benefit of the deployment. Perdomo et al.^25^ and Liley et al.^26^ study the setting of performing successive model updates, each time after deploying the previous model for decision-making. Perdomo et al.^25^ study when, over successive deployments, the predictive performance stabilizes or reaches optimality, and Liley et al.^26^ study both model stability and the effect of model deployment on outcomes. Our work may be seen as a special case of these works with only a single model deployment and no model update, but we add new insights as we describe exactly *when* a single model deployment leads to harm and good post-deployment discrimination.

Several groups have studied out-of-distribution generalization and its connections to causality and invariance^27^^,^^28^^,^^29^ with the aim of removing a model’s dependency on spurious correlations. Again, our work differs, as we are interested in characterizing model performance following a very specific distribution change (a treatment policy change induced by a prediction model), and our main concern is the effect of this policy change on outcomes. Finally, current guidelines on prediction model validation and deployment focus on discrimination and calibration only, not on these newer invariance metrics.^5^^,^^22^ Concurrent work studies the same setup as ours through the lens of domain adaptation, where each (pre-)deployment setting is formalized as a domain.^30^ They describe when the effect of deploying (or updating) an OPM for decision support can be estimated without observing outcomes under the target domain; however, both their assumptions and results diverge from the present work.

We are not the first to warn against naively using OPMs for decision support (see, e.g., points 6.3 and 6.7 in Assel et al.^31^). However, (intended) misuse of OPMs is still far too common in medical research and guidelines, and the reason why this can lead to harmful situations is not well understood. Our work provides a formal approach to understanding the risks of using OPMs without proper validation.

## Discussion

We showed when OPMs yield harmful self-fulfilling prophecies, meaning they lead to patient harm when used for treatment decision-making but retain good discrimination after deployment. Moreover, we showed that when a model is well calibrated before and after deployment, it is not useful for treatment decision-making. The upshot of these findings is that not only do harmful and self-fulfilling policies exist, but in some scenarios, it is even *desirable* to see worse discrimination after deployment, as this may signal a beneficial new policy in terms of patient outcomes. These results demonstrate the inadequacy of evaluating predictive models post-deployment with discrimination and calibration when these models are used for decision-making.

When interpreting the performance of an OPM post-deployment, a “high AUC is good, low AUC is bad” mindset proves to be too simplistic. A higher performance post-deployment does not necessarily indicate a beneficial policy change, and a lower performance post-deployment is not, by itself, a sign that the model is harmful. For instance, the latter may be due to poor generalization performance but may also be due to the OPM implementation being beneficial and changing the population so that the prediction task becomes harder (hence a lower AUC), shedding a new light on results such as those of Wong et al.^32^ In this second circumstance, removing an OPM-based policy due to low performance would, in fact, be detrimental in terms of patient outcomes. When presented with a case where an OPM was deployed and re-evaluated for predictive accuracy post-deployment, our Table 1 can provide concrete guidance for determining whether the new policy was harmful or beneficial. In short, the pre-existing treatment policy, the interpretation of the outcome variable, and the change in AUC post-deployment can already give an indication of the effect of the new policy on patient outcomes, provided the assumptions of our settings hold.

In recent years, the United States Food and Drug Administration (FDA) and the European Medical Agency (EMA) have been developing protocols on regulating AI-based software for medical applications. The FDA’s guiding principles explicitly include a total product life-cycle approach, where post-deployment monitoring and certain potential model updates are foreseen and described during initial approval, both with the aim to ensure post-deployment safety, for example, under dataset shifts but also to avoid the need for re-approval after each model update. Though their guiding principles on “good machine learning practice”^33^ and “predetermined change control plans”^34^ both mention post-deployment monitoring for safety, the intended monitoring seems to center mostly around predictive performance, which our results demonstrate to be insufficient to protect against harmful self-fulfilling prophecies. The EMA’s “Reflection paper on the use of artificial intelligence in the life cycle of medicines” also recommends pre-planned monitoring but only of predictive performance.^35^

Requiring explicit monitoring of changes in patient outcomes over time and changes in treatment policy may, in some cases, be warranted. Though monitoring patient outcomes in important pre-determined patient subgroups before and after deployment may detect harmful model deployments, before-after comparisons are plagued by well-known biases such as potential concurrent changes in other policies or general time trends in outcomes. The best experiment to demonstrate the safety of deploying an OPM is to conduct a cluster RCT, where some caregivers are randomly selected to have access to the OPM and others are not. The difference in average outcomes of patients between the caregivers with and without access determines whether using the OPM led to better patient outcomes. When cluster randomized trials are unfeasible, other, smaller clinical studies might be the next best option.^36^^,^^37^ How to pre-specify safe model monitoring and updates after deployment in a total product life-cycle approach is left for future work.

Finally, we note that developing OPMs that ignore the historic treatment policy is, in many cases, a bad approach when the ultimate aim is to improve the policy for assigning treatments.^8^^,^^38^ Instead, researchers should consider using methods developed for improving decisions, such as prediction-under-intervention models or models of the conditional average treatment effect (CATE) (for example, Feuerriegel et al.,^39^ Wager and Athey,^40^ and Kreif et al.^41^). These methods require either the availability of large, good-quality RCTs with detailed information on pre-treatment patient characteristics or observational datasets where the assumption of no unobserved confounding is tenable. Also, these methods require a specific evaluation strategy, as they are evaluated on their ability to predict outcomes under a new treatment policy or the effect of introducing a new treatment policy on outcomes, not on their ability to predict the outcome under the historical treatment policy. For these models, cluster randomized trials are also the gold standard for evaluating the effect of the new treatment policy on patient outcomes, but other, specialized evaluation methods exist.^37^^,^^42^ In this light, evaluating an OPM-based policy post-deployment with Table 1 is not a recommended practice for new OPMs but rather a way to determine post hoc whether potential harm was done when an OPM was deployed for decision support without the proper prior evaluation.

Some limitations remain, encoded in the assumptions of our formal results. The setting we describe is kept simple on purpose, a choice that helps to pinpoint the problem but somewhat limits the applicability of this theory to real-world use cases. The extension of our results to other feature types (continuous and categorical X), non-threshold-based policies, or a $\pi_{0}$ that is not constant (i.e., varies with X) or is non-deterministic is left to future work. Other, more complex use cases worth investigating might display policies that are harmful to subgroups identified by variables not included in the list of predictors of the model. The continuation of this line of work entails the re-evaluation of the metrics to monitor and assess a model’s effectiveness, and given that model deployments for decision support are interventions, this will benefit from using the language of causal inference.

### Conclusion

OPMs can yield harmful self-fulfilling prophecies when used for decision-making. The current paradigm on prediction model development, deployment, and monitoring needs to shift its primary focus away from predictive performance and instead toward changes in treatment policy and patient outcomes.

## Resource availability

### Lead contact

Requests for further information and resources should be directed to and will be fulfilled by the lead contact, Wouter A.C. van Amsterdam, (w.a.c.vanamsterdam-3@umcutrecht.nl).

### Materials availability

This study did not generate new unique reagents.

### Data and code availability

• No data were used in this study except those generated in the numerical experiments.
• The code to reproduce the numerical experiments is deposited at Zenodo.^43^
• Any additional information required to reproduce the results reported in this paper is available from the lead contact upon request.

## Author contributions

Study conception and design, W.A.C.v.A. and G.C.; writing – original draft, W.A.C.v.A. and G.C.; writing – review & editing, all authors.

## Declaration of interests

The authors declare no competing interests.

## Declaration of generative AI and AI-assisted technologies in the writing process

During the preparation of this work, the authors used ChatGPT in order to shorten the summary and the bigger picture section. After using this tool, the authors reviewed and edited the content as needed and take full responsibility for the content of the publication.

## Supplemental information

Document S1. Figures S1–S6, Tables S1–S3, and Notes S1–S3

Document S2. Article plus supplemental information
