# Supplementary material for: When accurate prediction models yield harmful self-fulfilling prophecies
Source: Patterns (N Y). 2025 Apr 11;6(4):101229. doi: 10.1016/j.patter.2025.101229 (PMC12010445; doi:10.1016/j.patter.2025.101229)
Supplement: Document S1. Figures S1–S6, Tables S1–S3, and Notes S1–S3 [file mmc1.pdf]

Patterns, Volume *6*

Supplemental information

When accurate prediction models

yield harmful self-fulfilling prophecies

Wouter A.C. van Amsterdam, Nan van Geloven, Jesse H. Krijthe, Rajesh Ranganath, and Giovanni Cinà

Supplemental Note S1 Hypothetical example of a harmful self-fulfilling prophecy

We now give a full-fledged hypothetical example based on realistic assumptions that would result in an OPM yielding a policy that is both harmful and self-fulfilling.

Consider the problem of selecting a subset of end-stage cancer patients for palliative radiotherapy. Such treatment has severe side-effects and thus domain experts advise to attempt to reduce over-treatment in the population of cancer patients. To comply with this advice, a medical center needs to decide which patients will not be eligible anymore for the therapy.

The medical center decides to give the therapy to patients with the longest expected overall survival, under the assumption that these patients would be those for whom the side-effects are justifiable. To support this policy, researchers built an OPM to predict the probability of 6-months overall survival based on pre-treatment tumor growth rate using historical patient records from the medical center. Fast-growing tumors are more aggressive so these patients have a shorter survival overall. The medical center decides to use this model to allocate the therapy and tests the model's discrimination post deployment. Based on this we have the following facts:

1. $X = 1$: fast growing tumor, $X = 0$: slow-growing tumor;
2. $\pi_0(X) = 1$, the historical policy was treating everyone;
3. $p(Y_0 = 1|X = 0) - p(Y_0 = 1|X = 1) > 0$, with radiotherapy, patients with fast growing tumors live shorter

A model with a good fit to the data will predict that patients with slow-growing tumors have a higher probability of 6-months survival. We also assume that the new policy is non-constant and favors those with highest predicted outcome, which means that the new policy will be 'treat patients with slow growing tumors but not those with fast growing tumors':

$$\pi_f(X) = 1 - X$$

However, it is well known that fast-growing tumors respond better to radiotherapy than slow growing tumors[15]. Based on this we add the following two assumptions:

1. $p(Y_0 = 1|X = 0) - p(Y_1 = 1|X = 0) = 0$, radiotherapy is not effective against slow growing tumors;
2. $\delta := p(Y_0 = 1|X = 1) - p(Y_1 = 1|X = 1) < 0$, radiotherapy *is* effective for fast growing tumors.

This means that the antecedent of Proposition 5 is satisfied, meaning that f yields a self-fulfilling prophecy in combination with any threshold λ such that the resulting policy is non-constant. Removing the therapy from the group $X = 1$ will worsen their outcomes by δ, separating the two groups even more and resulting in higher AUC post-deployment.

Moreover, according to the first case of Proposition 7, the OPM is harmful because the new treatment policy leads to worse outcomes for the group with fast growing tumors ($X = 1$). So the OPM-based policy treats exactly the wrong patients: those who do not benefit from treatment still receive it, those who would benefit from treatment do not, but paradoxically it has good discrimination before and after deployment.

Supplemental Note S2 Proofs of main results

Proof of Proposition 5.

Proof

First we give some elementary definitions and equalities. Define

$$\mu_i(x) = p_i(Y = 1|X = x) = (1 - \pi_i(x))\, p(Y_0 = 1|X = x) + \pi_i(x)p(Y_1 = 1|X = x) \tag{12}$$

So by the law of total probability we can write

$$p_i(Y = 1) = p_i(X = 0)\mu_i(0) + p_i(X = 1)\mu_i(1) \tag{13}$$

By Bayes rule we have:

$$p_i(X = x|Y = y) = \frac{p_i(Y = y|X = x)p(X = x)}{p_i(Y = y)} \tag{14}$$

Filling in the definition of $\mu_i(x)$ into 14 using the assumption that $p_i(X = x) = p(X = x)$ we have in particular:

$$p_i(X = x|Y = 1) = \frac{\mu_i(x)p(X = x)}{p_i(Y = 1)} \tag{15}$$

ROC-curves are created by transforming a continuous-valued function to a binary prediction based on a varying *threshold* τ and calculating the *sensitivity* and *specificity* for each value of τ:

$$\text{sensitivity} = p(f(X) \geq \tau|Y = 1) \tag{16}$$

$$\text{specificity} = p(f(X) < \tau|Y = 0) \tag{17}$$

For each possible threshold, all predictions under the threshold are labeled *negative* and all predictions greater or equal to the threshold *positive*. In the case of a binary X, $f(X)$ only takes two unique values so the ROC-curve is given by just three points:

1. sensitivity $= 1$, specificity $= 0$ ($\tau = -\infty$)
2. sensitivity $= 0$, specificity $= 1$ ($\tau = +\infty$)
3. sensitivity $=$ sens, specificity $=$ spec ($\tau = \max_X f(X)$)

See Figure S1. We can directly calculate the AUC by dividing the area under the ROC-curve in two adjacent non-overlapping triangles. This gives us the following expression for the AUC (see also[43]):

$$\text{AUC} = \frac{1}{2}\text{sens} + \frac{1}{2}\text{spec} \tag{18}$$

In this binary case, the area-under the ROC curve is thus determined by a single point denoted as (spec,sens). A pair (f, λ) is self-fulfilling when:

$$\text{AUC}(f) - \text{AUC}(0) = \frac{1}{2}\left(\text{sens}_f + \text{spec}_f - \text{sens}_0 - \text{spec}_0\right) \geq 0 \tag{19}$$

We structure the proof by first creating an enumeration over all possible scenarios. We assumed π_f is non-constant, which implies that f varies with X. Since X is binary, it must be that either $f(0) > f(1)$ or $f(1) > f(0)$. These cases are symmetric under relabeling of X so without loss of generality we proceed assuming that $f(0) > f(1)$ is the case. Since π_f is not constant but π_0 is, it must be that either the treatment policy changes for $X = 0$ but remains the same for $X = 1$, or vice versa. This in turn implies that either $\mu_f(0) = \mu_0(0)$ or $\mu_f(1) = \mu_0(1)$.

To provide a proof for the theorem, we enumerate all the subcases based on two factors:

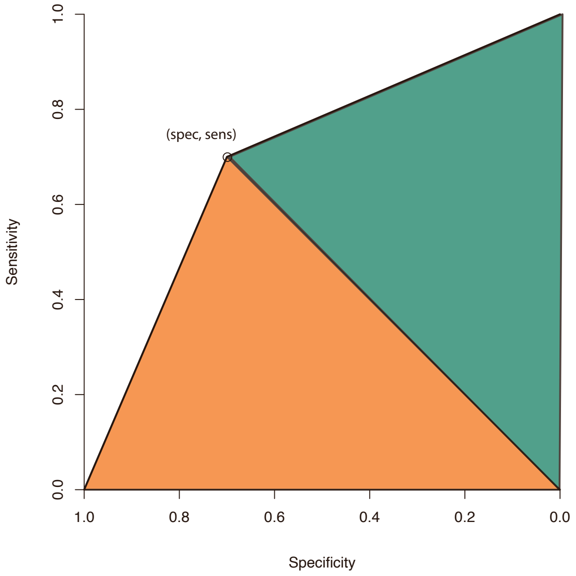

Figure S1: AUC for a binary predictor X

1. for which group does the policy change ($X = 0$ or $X = 1$)?
2. for the group with the policy change, does the outcome under the new policy remain the same (the policy is inconsequential as the treatment effect is zero), increase or decrease (this will be beneficial or detrimental depending on whether $Y = 1$ is good or bad)

This leads to the following 6 cases:

- policy change for which X?

   0. $\pi_f(0) \neq \pi_0(0)$
      effect of policy change:
      =: $\mu_f(0) = \mu_0(0)$, $\mu_f(1) = \mu_0(1)$
      <: $\mu_f(0) < \mu_0(0)$, $\mu_f(1) = \mu_0(1)$
      >: $\mu_f(0) > \mu_0(0)$, $\mu_f(1) = \mu_0(1)$
   1. $\pi_f(1) \neq \pi_0(1)$
      effect of policy change:
      =: $\mu_f(0) = \mu_0(0)$, $\mu_f(1) = \mu_0(1)$
      <: $\mu_f(0) = \mu_0(0)$, $\mu_f(1) < \mu_0(1)$
      >: $\mu_f(0) = \mu_0(0)$, $\mu_f(1) > \mu_0(1)$

These 6 combinations cover all possibilities. Since we have that $f(0) > f(1)$, by assumption of a non-deterministic $\pi_f(x) = I_{f(x)>\lambda}$ it must be that for all subcases $\pi_f(0) = 1$ and $\pi_f(1) = 0$. Each of these cases have implications for π_0 and, depending on which policy changes, $p(Y_1 = 1|X = 0) - p(Y_0 = 1|X = 0)$ or $p(Y_1 = 1|X = 1) - p(Y_0 = 1|X = 1)$. For instance case $(0, >)$ specifies that $\pi_f(0) \neq \pi_0(0)$. Because $\pi_f(0) = 1$, it follows that $\pi_0 = 0$. And because $Y_1(0) = \mu_f(0) > \mu_0(0) = Y_0(0)$ it must be that $p(Y_1 = 1|X = 0) - p(Y_0 = 1|X = 0) > 0$, meaning that the treatment increases the outcome for the group with $X = 0$.

In the two cases where the outcomes do not change ($(0, =)$ and $(1, =)$), (f, λ) is trivially self-fulfilling as nothing changes in the distribution of X, Y so the sensitivity and specificity remain the same.

We first prove self-fulfillingness in cases $(0, >)$ and $(0, <)$:

Case $(0,>)$ **and** $(0,<)$ We first address case $(0,>)$, which gives us this information:

- $\pi_f(0) \neq \pi_0(0)$
- $\mu_f(0) > \mu_0(0)$
- $\mu_f(1) = \mu_0(1)$

Since $f(0) > f(1)$ we get these sensitivity and specificity:

$$\text{sens}_i = p_i(f(X) \geq \max(f)|Y=1) = p_i(X=0|Y=1) \tag{20}$$
$$\text{spec}_i = p_i(f(X) < \max(f)|Y=0) = p_i(X=1|Y=0) \tag{21}$$

with $i \in \{0, f\}$. Plugging this into 19 yields:

$$\begin{aligned}\text{AUC}(f) - \text{AUC}(0) &= \frac{1}{2}\big(p_f(X=0|Y=1) - p_0(X=0|Y=1) \\ &\quad + p_f(X=1|Y=0) - p_0(X=0|Y=0)\big) \\ &= \frac{1}{2}\big(\mu_f(0)\frac{p(X=0)}{p_f(Y=1)} - \mu_0(0)\frac{p(X=0)}{p_0(Y=1)} \\ &\quad + (1-\mu_f(1))\frac{p(X=1)}{p_f(Y=0)} - (1-\mu_0(1))\frac{p(X=1)}{p_0(Y=0)}\big)\end{aligned}$$

where the first equality is by substitution and rearrangement, and the second by Bayes rule. We can determine the sign of this difference based on the sign of two terms:

$$= \frac{1}{2}\big(p(X=0)\big(\frac{\mu_f(0)}{p_f(Y=1)} - \frac{\mu_0(0)}{p_0(Y=1)}\big) \tag{22}$$
$$+ p(X=1)\big(\frac{1-\mu_f(1)}{p_f(Y=0)} - \frac{1-\mu_0(1)}{p_0(Y=0)}\big)\big) \tag{23}$$

We write the difference between pre- and post-deployment expected outcome for the group $X = 0$ as

$$\delta := \mu_f(0) - \mu_0(0) \tag{24}$$

This gives us

$$p_f(Y=1) = p(X=1)\mu_f(1) + p(X=0)\mu_f(0) \tag{25}$$
$$= p(X=1)\mu_0(1) + p(X=0)(\mu_0(0) + \delta) \tag{26}$$
$$= p_0(Y=1) + p(X=0)\delta \tag{27}$$

where the first step is the law of total probability, the second by the definition of δ and the case information $\mu_f(1) = \mu_0(1)$, and finally again using the law of total probability. Furthermore

$$p_f(Y=0) = 1 - p_f(Y=1) \tag{28}$$
$$= 1 - p_0(Y=1) - p(X=0)\delta \tag{29}$$
$$= p_0(Y=0) - p(X=0)\delta \tag{30}$$

where the second step is by our previous calculation and the other two just the property of binary outcomes. We can now determine the signs of the two terms in 22.

$$\text{sign}\Big[\frac{\mu_f(0)}{p_f(Y=1)} - \frac{\mu_0(0)}{p_0(Y=1)}\Big] = \text{sign}\Big[\frac{\mu_f(0)p_0(Y=1) - \mu_0(0)p_f(Y=1)}{p_f(Y=1)p_0(Y=1)}\Big] \tag{31}$$

$$= \text{sign}\big[\mu_f(0)p_0(Y=1) - \mu_0(0)p_f(Y=1)\big] \tag{32}$$

The first equality is cross-multiplying, the second equality is because the product of two probabilities (which are positive by assumption) is always a positive number.

Filling in the definition of δ :

$$\text{sign}\Big[\frac{\mu_f(0)}{p_f(Y=1)} - \frac{\mu_0(0)}{p_0(Y=1)}\Big] \tag{33}$$

$$= \text{sign}\big[(\mu_0(0)+\delta)p_0(Y=1) - \mu_0(0)(p_0(Y=1) + p(X=0)\delta)\big] \tag{34}$$

$$= \text{sign}\big[\delta p_0(Y=1) - \mu_0(0)p(X=0)\delta\big] \tag{35}$$

$$= \text{sign}\big[\delta(p_0(Y=1) - \mu_0(0)p(X=0))\big] \tag{36}$$

$$= \text{sign}\big[\delta\mu_0(1)p(X=1)\big] \tag{37}$$

$$= \text{sign}\big[\delta\big] \tag{38}$$

In the second equality we remove canceling terms. In the third equality we pull out δ. In the fourth equality we use the expansion of $p_0(Y=1) = p(X=0)\mu_0(0) + p(X=1)\mu_0(1)$, and for the final equation we note again that $\mu_0(1)$ and $p(X=1)$ are positive probabilities so the sign is determined by the sign of δ.

Now for the second term of 22:

$$\text{sign}\Big[\frac{1-\mu_f(1)}{p_f(Y=0)} - \frac{1-\mu_0(1)}{p_0(Y=0)}\Big] = \text{sign}\Big[\frac{1-\mu_0(1)}{p_f(Y=0)} - \frac{1-\mu_0(1)}{p_0(Y=0)}\Big] \tag{39}$$

$$= \text{sign}\Big[\big(1-\mu_0(1)\big)\Big(\frac{1}{p_f(Y=0)} - \frac{1}{p_0(Y=0)}\Big)\Big] \tag{40}$$

$$= \text{sign}\Big[\frac{1}{p_f(Y=0)} - \frac{1}{p_0(Y=0)}\Big] \tag{41}$$

$$= \text{sign}\Big[\frac{p_0(Y=0) - p_f(Y=0)}{p_f(Y=0)p_0(Y=0)}\Big] \tag{42}$$

$$= \text{sign}\big[p_0(Y=0) - p_f(Y=0)\big] \tag{43}$$

$$= \text{sign}\big[p_0(Y=0) - p_0(Y=0) + p(X=0)\delta\big] \tag{44}$$

$$= \text{sign}\big[p(X=0)\delta\big] \tag{45}$$

$$= \text{sign}\big[\delta\big] \tag{46}$$

The first equality uses the case assumption that $\mu_f(1) = \mu_0(1)$. The second equality pulls out the common term $(1-\mu_0(1))$. The third equality follows because $0 < \mu_0(1) < 1$. The fourth and fifth equality are cross-multiplying and again using the positive probability property. In the sixth equality we substitute in the definition of δ. The seventh equality removes the canceling terms, and the final equality again relies on that $0 < p(X=0)$.

So both terms in 22 have the sign of δ. In subcase $(0,>)$ δ has positive sign, so

$$\text{AUC}(f) - \text{AUC}(0) > 0$$

and (f, λ) is self-fulfilling.

Immediately it is clear that in subcase $(0,<)$, (f,λ) is not self-fulfilling, as subcase $(0,<)$ equals subcase $(0,>)$ in all respects except that instead it has a negative sign for δ.

Case $(1, >)$ **and** $(1, <)$ We first address case $(1, >)$, which gives us this information:

- $\pi_f(1) \neq \pi_0(1)$
- $\mu_f(0) = \mu_0(0)$
- $\mu_f(1) > \mu_0(1)$

Again we write the difference between pre- and post-deployment expected outcome as δ, this time for the group $X = 1$:

$$\delta := \mu_f(1) - \mu_0(1) \tag{47}$$

This gives us

$$\begin{aligned} p_f(Y = 1) &= p(X = 1)\mu_f(1) + p(X = 0)\mu_f(0) && (48)\\ &= p(X = 1)(\mu_0(1) + \delta) + p(X = 0)\mu_0(0) && (49)\\ &= p_0(Y = 1) + p(X = 1)\delta && (50) \end{aligned}$$

where the first step is the law of total probability, the second by the definition of δ and the case information $\mu_f(0) = \mu_0(0)$, and finally again using the law of total probability. Furthermore

$$\begin{aligned} p_f(Y = 0) &= 1 - p_f(Y = 1) && (51)\\ &= 1 - p_0(Y = 1) - p(X = 1)\delta && (52)\\ &= p_0(Y = 0) - p(X = 1)\delta && (53) \end{aligned}$$

where the second step is by our previous calculation and the other two just the property of binary outcomes. We can now determine the signs of the two terms in 22.

The first two steps for the first are the same as in the case $(0, >)$ (see Equation 31), after these steps we substitute in the new definition of δ:

$$\begin{aligned} &\text{sign}\Big[\frac{\mu_f(0)}{p_f(Y = 1)} - \frac{\mu_0(0)}{p_0(Y = 1)}\Big] && (54)\\ &= \text{sign}\big[\mu_f(0)p_0(Y = 1) - \mu_0(0)p_f(Y = 1)\big] && (55)\\ &= \text{sign}\big[\mu_0(0)p_0(Y = 1) - \mu_0(0)(p_0(Y = 1) + p(X = 1)\delta)\big] && (56)\\ &= \text{sign}\big[-\mu_0(0)p(X = 0)\delta\big] && (57)\\ &= \text{sign}\big[-\delta\big] && (58) \end{aligned}$$

In the third equality we remove canceling terms. For the final equation we note again that $\mu_0(0)$ and $p(X = 0)$ are positive probabilities so the sign is determined by the sign of δ.

Now for the second term of 22:

subcase		π_0	$\pi_f(0)$	$\pi_f(1)$	CATE(0)	CATE(1)	self-fulfilling
0	=	0	1	0	0		yes
0	<	0	1	0	-		no
0	>	0	1	0	+		yes
1	=	1	1	0		0	yes
1	<	1	1	0		+	yes
1	>	1	1	0		-	no

Table S1: Enumeration of all possible subcases. The first column indicates for which value of X the treatment policy changes. The second column indicates whether this change improves outcomes for that group (>), reduces outcomes (<) or is irrelevant (=). $+/-$ indicates the sign of the subgroup treatment effect $\text{CATE}(x) := p(Y_1 = 1|X = x) - p(Y_0 = 1|X = x)$;

$$\text{sign}\Big[\frac{1-\mu_f(1)}{p_f(Y=0)} - \frac{1-\mu_0(1)}{p_0(Y=0)}\Big] \tag{59}$$

$$= \text{sign}\Big[\frac{(1-\mu_f(1))p_0(Y=0) - (1-\mu_0(1))p_f(Y=0)}{p_f(Y=0)p_0(Y=0)}\Big] \tag{60}$$

$$= \text{sign}\big[(1-\mu_f(1))p_0(Y=0) - (1-\mu_0(1))p_f(Y=0)\big] \tag{61}$$

$$= \text{sign}\big[(1-(\mu_0(1)+\delta))p_0(Y=0) - (1-\mu_0(1))(p_0(Y=0) - p(X=1)\delta)\big] \tag{62}$$

$$= \text{sign}\big[-\delta p_0(Y=0) - (1-\mu_0(1))(-p(X=1)\delta)\big] \tag{63}$$

$$= \text{sign}\big[-\delta(p_0(Y=0) - (1-\mu_0(1))p(X=1))\big] \tag{64}$$

$$= \text{sign}\big[-\delta((1-\mu_0(0))p(X=0))\big] \tag{65}$$

$$= \text{sign}\big[-\delta\big] \tag{66}$$

The first equality uses cross-multiplication to gather the sum. The second equality follows because we're dividing by a positive number. The third equality is filling in the definition on δ. The fourth equality removes canceling terms. The fifth equality factors out $-\delta$. The seventh equality is by the law of total probability.

So both terms in 22 have the sign of $-\delta$. In subcase $(1, >)$ δ has positive sign, so

$$\text{AUC}(f) - \text{AUC}(0) < 0$$

and (f, λ) is not self-fulfilling.

Immediately it is clear that in subcase $(1, <)$, (f, λ) is self-fulfilling, as subcase $(1, <)$ equals subcase $(1, >)$ in all respects except that instead it has a negative sign for δ.

Enumerating all the cases As said, in the two cases where the outcomes do not change $((0, =), (1, =))$, (f, λ) is trivially self-fulfilling.

Putting all the pieces of information for all subcases together in Table S1 we see that when $p(Y_1 = 1|X = x) - p(Y_0 = 1|X = x) \geq 0$ (the treatment effect is never negative), (f, λ) is self-fulfilling. Also, when $p(Y_1 = 1|X = x) - p(Y_0 = 1|X = x) < 0$ (the treatment effect is always negative), (f, λ) is never self-fulfilling. These observations conclude the proof.

■

Proof of Proposition 7.

Given that we assumed binary T and X, we can write the expected value of the outcome conditional on these two variables with four parameters without making parametric assumptions, marginalizing over other

variables different than X and T. For ease of interpretation of our results we write the expected value as a sum:

$$p(Y_{T=t} = 1|X = x) = \alpha + \beta_x x + \beta_t t + \beta_{xt} xt \tag{67}$$

Note that this is not an assumption on the generating process of the outcome Y, which could have arbitrary form, it is only a formal device to represent the four outcomes of interest, one for each value of X and T.

We now proceed to prove the Proposition for the case where higher outcome is better; to obtain a proof for the symmetric case (higher outcome is worse) one needs only to switch the sign in the inequalities 68 and 69, along with their specialization in the subcases.

Proof A treatment is harmful for the group with $X = x'$ iff $p_f(Y = 1|X = x') < p_0(Y = 1|X = x')$, where according to definition 3 $p_i(Y = 1|X) = \mathbb{E}_{T\sim\pi_i(X)}\, p(Y_T = 1|X)$ The proof continues as a case distinction depending on the value of x'.

Case $x' = 1$**.** For $x' = 1$ the definition of harmful translates to

$$(\pi_f(1) - \pi_0(1))\,(\beta_t + \beta_{xt}) < 0 \tag{68}$$

We consider the possible values of π_f and π_0 in subcases. Note that if $\pi_f(1) = \pi_0(1)$ the above inequality cannot hold since all terms cancel out and the treatment cannot be harmful (because nothing changes for group $X = 1$), so we only consider subcases where these two differ.

Subcase 1. We have $\pi_f(1) = 0, \pi_f(0) = 1$ and $\pi_0(x) = 1$. In this scenario, we were treating everyone and with the new policy we withhold treatment from group $X = 1$. In this case statement 68 specializes to $\beta_t + \beta_{xt} > 0$, meaning that treatment was beneficial and removing it will do damage to group $X = 1$.

Subcase 2. We have $\pi_f(1) = 1, \pi_f(0) = 0$ and $\pi_0(x) = 0$. In this scenario, we were treating nobody and with the new policy we introduce treatment for group $X = 1$. In this case statement 68 specializes to $\beta_t + \beta_{xt} < 0$, meaning that treatment is harmful and adding it damages group $X = 1$.

Case $x' = 0$**.** For $x' = 0$ the definition of harmful translates to

$$(\pi_f(0) - \pi_0(0))\,\beta_t < 0 \tag{69}$$

Again if $\pi_f(0) = \pi_0(0)$ the above inequality cannot hold since all terms cancel out and the treatment cannot be harmful (because nothing changes for group $X = 0$), so we only consider subcases where these two differ.

Subcase 1. We have $\pi_f(1) = 0, \pi_f(0) = 1$ and $\pi_0(x) = 0$. In this scenario, we were treating nobody and with the new policy we introduce treatment from group $X = 0$. In this case the statement 69 specializes to $\beta_t < 0$, which is what we intended to prove.

Subcase 2. We have $\pi_f(1) = 1, \pi_f(0) = 0$ and $\pi_0(x) = 1$. In this circumstance statement 69 specializes to $\beta_t > 0$. ■

Proof of Theorem 10.

By assumption f is calibrated on the historical data, so:

$$f(X = x) = p_0(Y = 1|X = x) = \mathbb{E}_{T\sim\pi_0(x)}\, p(Y_T = 1|X = x).$$

We now prove that f is calibrated on the deployment distribution generated by π_f iff for all $x \in \mathcal{X}$:

$$\pi_0(x) = \pi_f(x) \;\; or \;\; p(Y_1 = 1|X = x) = p(Y_0 = 1|X = x) \tag{70}$$

Proof As a shorthand define:

$$\mu_i(x) :=p_i(Y=1|X=x)$$
$$=(1-\pi_i(x))p(Y_0=1|X=x)+\pi_i(x)p(Y_1=1|X=x).$$

f is calibrated on the historical data so:

$$f(X=x)=\mu_0(x), \forall x \in \mathcal{X}. \tag{71}$$

By definition, f is calibrated on the post-deployment distribution when for all $\alpha \in [0,1]$ in the range of f, $\mathbb{E}_{X,Y\sim p_f(X,Y)}[Y|f(X)=\alpha]=\alpha$. So if f is calibrated on both the historic distribution and the post-deployment distribution we have that:

$$\begin{aligned}
&\mathbb{E}_{X,Y\sim p_f(X,Y)}[Y|f(X)=\alpha]\\
=&\,\mathbb{E}_{X,Y\sim p_f(X,Y)|f(X)=\alpha}[Y]\\
=&\,\mathbb{E}_{X,Y\sim p_f(X,Y)}[Y1[f(X)=\alpha]]/\,\mathbb{E}_{X\sim p_f(X)}[1[f(X)=\alpha]]\\
=&\,\mathbb{E}_{X,Y\sim p_0(X,Y)}[Y1[f(X)=\alpha]]/\,\mathbb{E}_{X\sim p_0(X)}[1[f(X)=\alpha]]
\end{aligned}$$

Where 1[..] is used for the indicator function. We first show that this holds iff for every $x \in \mathcal{X}$, $f(x)=\mu_0(x)=\mu_f(x)$. Note that in the last two equations above, the denominators are the same as $p_0(X)=p_f(X)$, so also the enumerators must be the same, so:

$$\begin{aligned}
&\mathbb{E}_{X\sim p_0(X)}\,\mathbb{E}_{Y\sim p_0(Y|X)}[Y1[f(X)=\alpha]]=\mathbb{E}_{X\sim p_f(X)}\,\mathbb{E}_{Y\sim p_f(Y|X)}[Y1[f(X)=\alpha]]\\
\iff&\mathbb{E}_{X\sim p_0(X)}\,1[f(X)=\alpha]\,\mathbb{E}_{Y\sim p_0(Y|X)}[Y]=\mathbb{E}_{X\sim p_f(X)}\,1[f(X)=\alpha]\,\mathbb{E}_{Y\sim p_f(Y|X)}[Y]\\
\iff&\mathbb{E}_{X\sim p_0(X)}\,1[f(X)=\alpha]\,\mathbb{E}_{Y_0,Y_1|X}[(1-\pi_0(X))Y_0+\pi_0(X)Y_1]\\
&=\mathbb{E}_{X\sim p_f(X)}\,1[f(X)=\alpha]\,\mathbb{E}_{Y_0,Y_1|X}[(1-\pi_f(X)Y_0+\pi_f(X)Y_1]
\end{aligned}$$

Since by assumption $p_0(X)=p_f(X)=p(X)$ we have that

$$\begin{aligned}
\iff&\mathbb{E}_{X\sim p(X)}\,1[f(X)=\alpha]\,\mathbb{E}_{Y_0,Y_1|X}[(1-\pi_0(X))Y_0+\pi_0(X)Y_1]\\
&=\mathbb{E}_{X\sim p(X)}\,1[f(X)=\alpha]\,\mathbb{E}_{Y_0,Y_1|X}[(1-\pi_f(X))Y_0+\pi_f(X)Y_1]\\
\iff&\mathbb{E}_{X,Y_0,Y_1}\,1[f(X)=\alpha]((1-\pi_0(X))Y_0+\pi_0(X)Y_1)\\
&=\mathbb{E}_{X,Y_0,Y_1}\,1[f(X)=\alpha]((1-\pi_f(X))Y_0+\pi_f(X)Y_1)\\
\iff&\mathbb{E}_X\,1[\mu_0(X)=\alpha]\mu_0(X)=\mathbb{E}_X\,1[\mu_0(X)=\alpha]\mu_f(X)
\end{aligned}$$

Where in the last line we substituted the definition of μ and used the assumption that $f(X)=\mu_0(X)$. Finally we note that by assumption $\pi_f(X)$ is non-constant. As X is binary it must be that f is injective. This implies that the expectation in the last line is given by the value of μ on a single point corresponding with α which proves that $\mu_0(X)=\mu_f(X)$.

Looking at the difference between $\mu_0(X)$ and $\mu_f(X)$ we see that:

$$\begin{aligned}
&\mu_f(X)-\mu_0(X)=\\
&\quad((1-\pi_f(X))p(Y_0=1|X)+\pi_f(X)p(Y_1=1|X))-\\
&\quad((1-\pi_0(X))p(Y_0=1|X)+\pi_0(X)p(Y_1=1|X))\\
&=(\pi_f(X)-\pi_0(X))\,(p(Y_1=1|X)-p(Y_0=1|X))
\end{aligned}$$

Hence the difference $\mu_f(X) - \mu_0(X)$ is zero iff at least one of the last two terms is zero. This means that f is calibrated on the deployment distribution iff for every x either $\pi_f(x) = \pi_0(x)$ or $p(Y_1 = 1|X = x) = p(Y_0 = 1|X = x)$

■

Supplemental Note S3 Numerical experiment

Experimental setup

We parameterize the joint distribution with a marginal distribution of X, a conditional of $T|X$ and $Y|T,X$, where we note that by assumption in the historic distribution, the treatment policy is *independent* of X, and also that the marginal distribution of X does not change after model deployment. Let $B(.)$ denote the bernoulli distribution and $\sigma(x) = \frac{1}{1+e^{-x}}$ the sigmoid (logistic) function.

$$x \sim B(p_x) \tag{72}$$
$$t = p_0(T) \in \{0, 1\} \tag{73}$$
$$\eta = \beta_0 + \beta_x x + \beta_t t + \beta_{xt} xt \tag{74}$$
$$y \sim B(\sigma(\eta)) \tag{75}$$

The parameter grid is:

parameter	distribution	interpretation	values
$p(X = 1)$	$p(X)$	marginal distribution of X	$0.2, 0.5$
$p_0(T = 1)$	$p_0(T)$	historic treatment policy	$0, 1$
β_0	$p(Y\|T, X)$	intercept on log odds scale	-0.5
β_x	$p(Y\|T, X)$	log odds ratio for X	$\log(1.1, 1.45, 1.8, 2.15, 2.5)$
β_t	$p(Y\|T, X)$	log odds ratio for T	log(1/2.5, 1/2.15, 1/1.8, 1/1.45, 1/1.1, 1, 1.1, 1.45, 1.8, 2.15, 2.5)
β_{xt}	$p(Y\|T, X)$	log odds for interaction between T and X	log(1/2.5, 1/2.15, 1/1.8, 1/1.45, 1/1.1, 1, 1.1, 1.45, 1.8, 2.15, 2.5)
higher Y		is higher Y better or worse	better, worse

Table S1: Parameters for numerical experiments

For these parameter values we first calculate the joint probability $p(X, Y)$ under the historic distribution. For some parameter values $p(Y = 1|X = 0) = p(Y = 1|X = 1)$, these settings are removed as they imply that the outcome risk is independent of X in the historical setting and would lead to a constant OPM-based treatment policy. When then also calculate outcomes under the new treatment policy with the outcome prediction model.

For each setting we calculate discrimination statistics (sensitivity, specificity, AUC), using:

$$\text{sens} = p(f(X) > \lambda|Y = 1) \tag{76}$$
$$\text{spec} = p(f(X) < \lambda|Y = 0) \tag{77}$$
$$\text{AUC} = \frac{1}{2}(\text{sens} + \text{spec}) \tag{78}$$

See Supplemental Note S2 for the derivation of the formula for AUC. First we need to determine whether the OPM-derived policy $\pi_f(X) = X$ (when $E[Y|X = 1] > E[Y|X = 0]$) or $\pi_f(X) = 1 - X$.

Then we compare the AUC before and after deployment to see if f is self-fulfilling, and the expected value of Y before and after deployment to see whether the new policy was harmful. Note that in our case harm for a subgroup is equivalent to marginal harm because the outcomes change for only one value of X (see Remark 9).

Results

.1 Experiment checks: Proposition 5 and Table 1

With these in hand, we can check Proposition 5 that: i) if the treatment effect is always positive, then (f, λ) is self-fulfilling; ii) if the treatment effect is always negative, then (f, λ) is not self-fulfilling.

sign(β_t)	sign($\beta_t + \beta_{xt}$)	not self-fulfilling (N)	self-fulfilling (N)
-1	-1	1508	0
-1	0	100	100
-1	1	200	200
0	-1	140	40
0	0	0	40
0	1	0	200
1	-1	320	44
1	0	0	180
1	1	0	1560

Table S2: Results of numerical experiments

We can also see that the numerical experiments follow Table 1.

higher Y is	$p_0(T = 1)$	selffulfilling	harmful (%)
worse	0	true	1
worse	0	false	0
worse	1	true	0
worse	1	false	1
better	0	true	0
better	0	false	1
better	1	true	1
better	1	false	0

Table S3: Results of numerical experiments, 2

.2 Plots

We now visualize the results. We include the following plots:

- Figure S2: A scatter plot of AUC on the historic data versus change in AUC in the post-deployment setting
- Figure S3: A scatter plot of the odds-ratio for treatment e^{β_t} for the group with $X = 0$, versus difference in AUC between pre and post deployment, with points colored by the treatment effect interaction term $e^{\beta_{xt}}$, and regions indicating whether the OPM-derived policy is harmful or not
- Figure S4: Like Figure S3 but with $e^{\beta_{xt}}$ on the x-axis and e^{β_t} as color codes.

Note that these three Figures include settings where the treatment is *always detrimental* (e.g. $\beta_t > 0, \beta_{xt} \geq 0$ and higher Y is worse). This means that no one should ever be treated with this treatment, making it highly unlikely that these treatments are in current clinical use so these settings are not very realistic. Instead, we subset the settings to those where the treatment is beneficial *on average*, although it does not have to be effective for both $X = 0$ and $X = 1$. This is typically the level of evidence available from RCTs before treatments are allowed on the market. These Figures are:

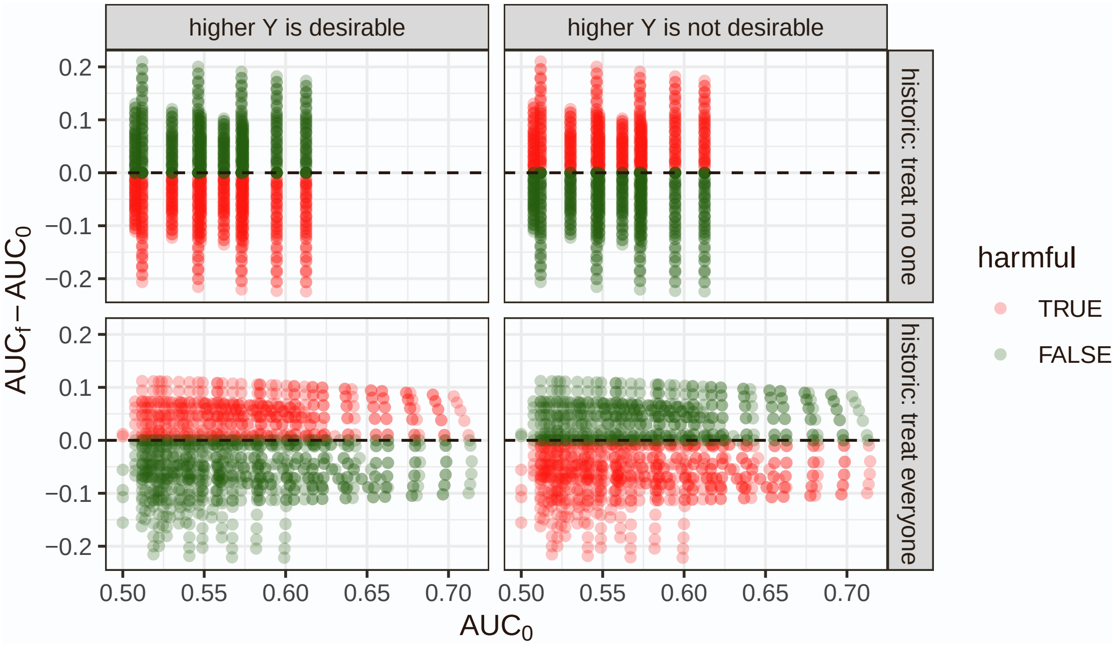

Figure S2: AUC under historic policy versus AUC increase under the policy with the outcome prediction model

- Figure S5: Like Figure S3 but subsetted to settings where treatment is are beneficial on average.
- Figure S6: Like Figure S4 but subsetted to settings where treatment is are beneficial on average.

Figure S5 is also presented in the main text.

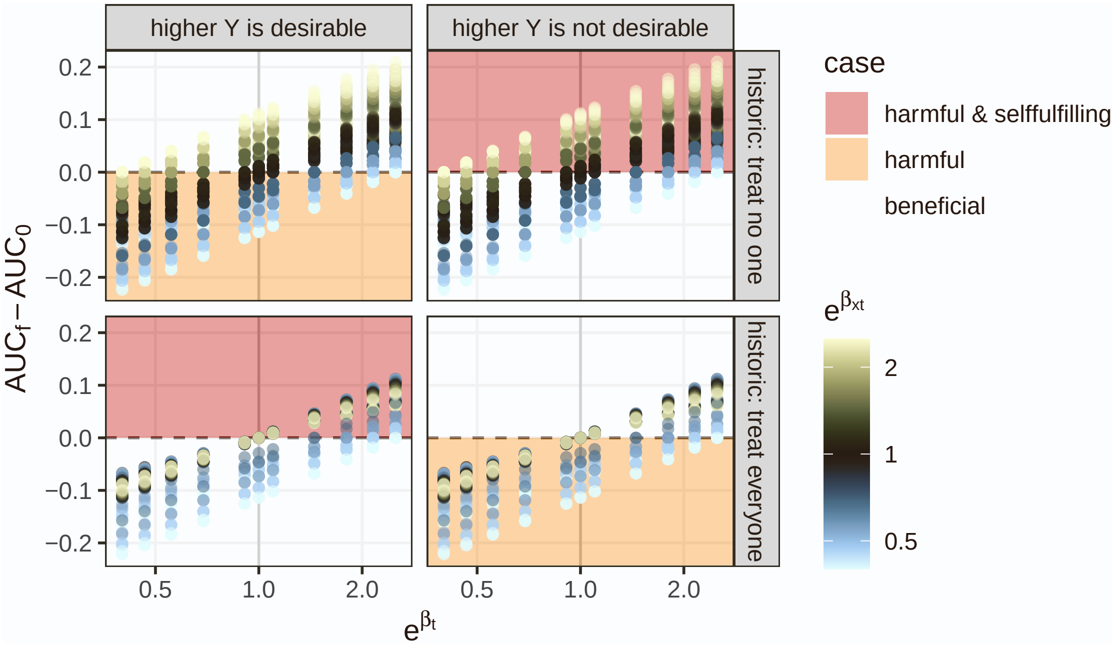

Figure S3: AUC difference versus treatment effect

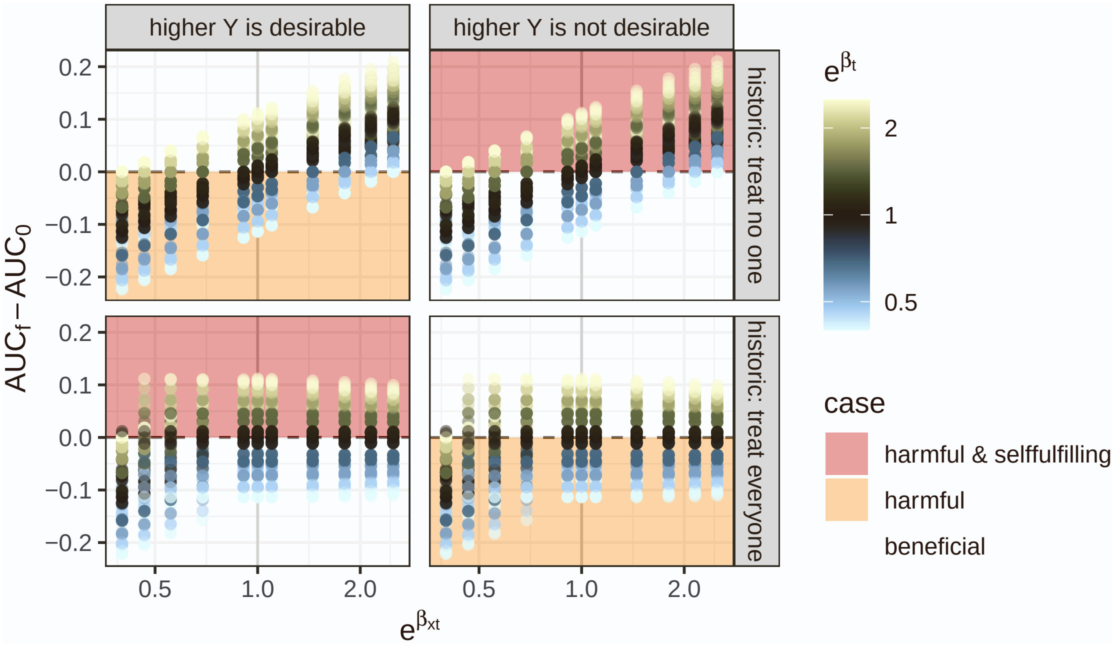

Figure S4: AUC difference versus treatment effect interaction

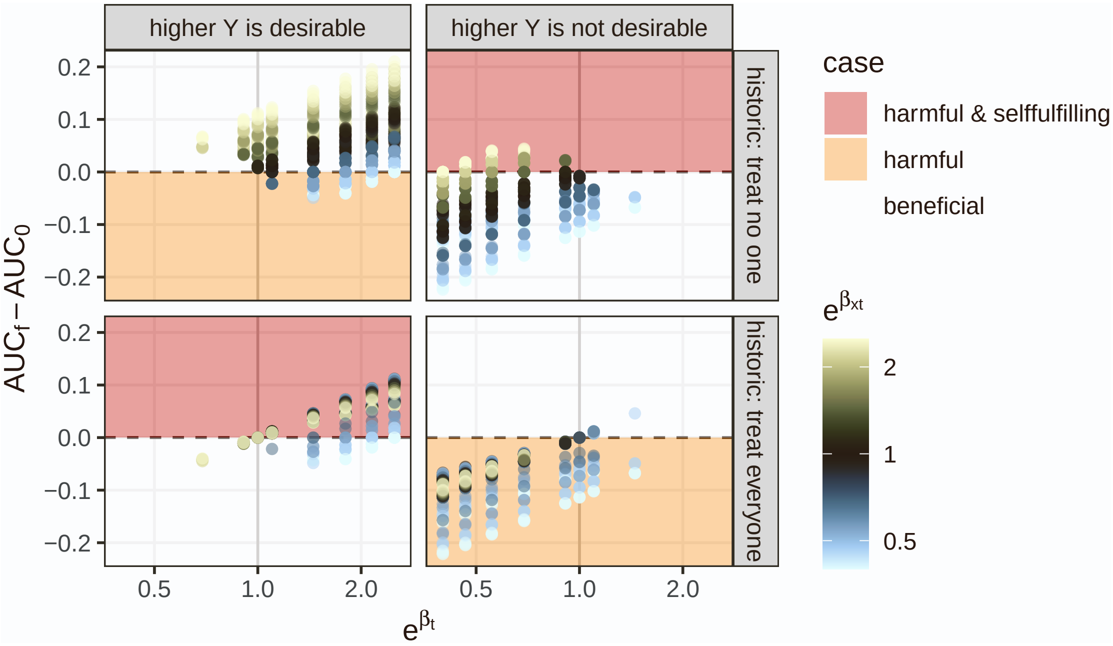

Figure S5: AUC difference versus treatment effect, only including settings where treatment is beneficial on average.

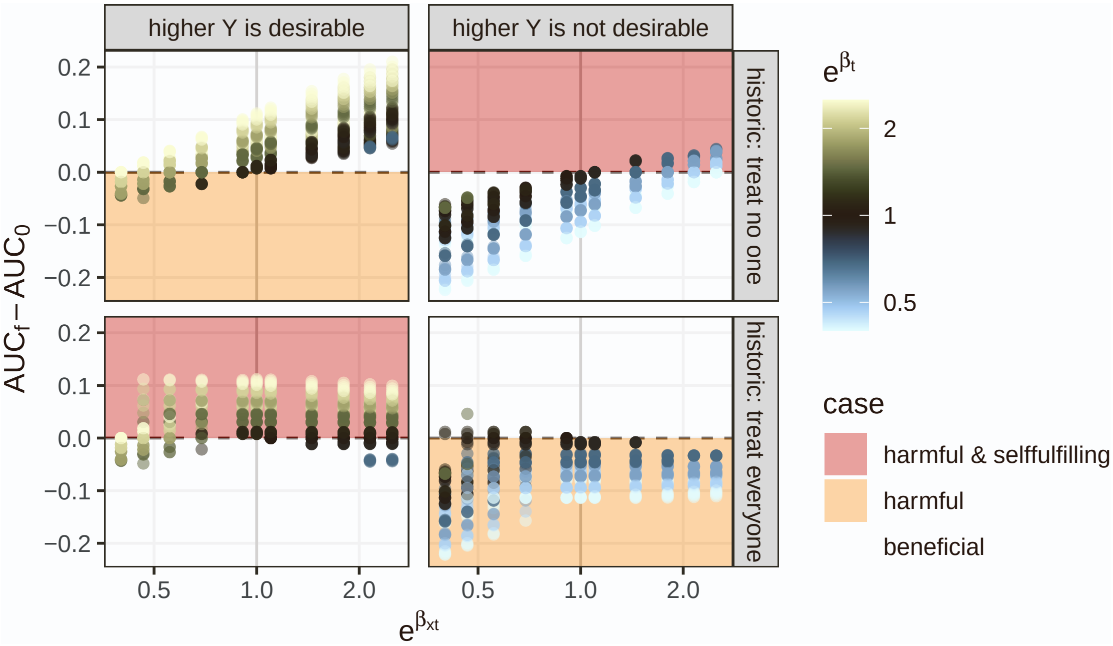

Figure S6: AUC difference versus treatment effect interaction, only including settings where treatment is beneficial on average.
