# Supplementary material for: When accurate prediction models yield harmful self-fulfilling prophecies
Source: Patterns (N Y). 2025 Apr 11;6(4):101229. doi: 10.1016/j.patter.2025.101229 (PMC12010445; doi:10.1016/j.patter.2025.101229)
Supplement: Document S2. Article plus supplemental information [file mmc2.pdf]

Article

Patterns

When accurate prediction models yield harmful self-fulfilling prophecies

Highlights

- Some prediction models yield harmful self-fulfilling prophecies when used for decisions
- These models harm patients but display good discrimination post-deployment
- Models that retain calibration post-deployment may be useless for decision-making
- The evaluation of prediction models for decision support must align with their final aim

Authors

Wouter A.C. van Amsterdam, Nan van Geloven, Jesse H. Krijthe, Rajesh Ranganath, Giovanni Cinà

Correspondence

w.a.c.vanamsterdam-3@umcutrecht.nl

In brief

Prediction models are often developed with the aim to guide treatment decisions, but can they cause harm? This study reveals that even models with good post-deployment performance—such as strong discrimination or calibration—can negatively impact patient outcomes. By identifying harmful self-fulfilling prophecies, the authors argue that evaluating prediction models should focus on their impact on treatment decisions and patient outcomes, not just predictive accuracy, to ensure they improve decision-making and patient care.

van Amsterdam et al., 2025, Patterns *6*, 101229
April 11, 2025 © 2025 The Author(s). Published by Elsevier Inc.
https://doi.org/10.1016/j.patter.2025.101229

CellPress

Patterns

CellPress
OPEN ACCESS

Article

When accurate prediction models yield harmful self-fulfilling prophecies

Wouter A.C. van Amsterdam,[1,8,9,*] Nan van Geloven,[2] Jesse H. Krijthe,[3] Rajesh Ranganath,[4] and Giovanni Cinà[5,6,7,8]
[1]Department of Data Science and Biostatistics, Julius Center for Health Sciences and Primary Care, University Medical Center Utrecht, University of Utrecht, Heidelberglaan 100, 3584 CX Utrecht, the Netherlands
[2]Department of Biomedical Data Sciences, Leiden University Medical Center, Albinusdreef 2, 2333 ZA Leiden, the Netherlands
[3]Pattern Recognition & Bioinformatics, Delft University of Technology, Mekelweg 5, 2628 CD Delft, the Netherlands
[4]Courant Institute of Mathematical Science, Department of Computer Science, Center for Data Science, New York University, 251 Mercer St., New York, NY 10012, USA
[5]Department of Medical Informatics, Amsterdam University Medical Center, Meibergdreef 9, 1105 AZ Amsterdam, the Netherlands
[6]Institute for Logic, Language and Computation, University of Amsterdam, Amsterdam, the Netherlands
[7]Pacmed, Amsterdam, the Netherlands
[8]These authors contributed equally
[9]Lead contact
*Correspondence: w.a.c.vanamsterdam-3@umcutrecht.nl
https://doi.org/10.1016/j.patter.2025.101229

THE BIGGER PICTURE To tailor treatment decisions to individual patients, many researchers develop prediction models. These models assess a patient's risk of an adverse outcome, such as a heart attack, based on their characteristics. Many believe that the best prediction models for decision-making are those that have the highest predictive performance, e.g., discrimination—the ability to assign higher risks to patients with the outcome compared to those without. Common advice is to keep evaluating discrimination after the model's implementation to ensure effective decision-making. We show, through a clinical example and mathematical proofs, that this belief is flawed because of the existence of so-called "harmful self-fulfilling prophecies": prediction models that retain good discrimination after implementation and yet harm patients when used for decision-making. The takeaway is that rather than relying on discrimination, we should assess models based on their impact on treatment decisions and patient outcomes.

SUMMARY

Prediction models are popular in medical research and practice. Many expect that by predicting patient-specific outcomes, these models have the potential to inform treatment decisions, and they are frequently lauded as instruments for personalized, data-driven healthcare. We show, however, that using prediction models for decision-making can lead to harm, even when the predictions exhibit good discrimination after deployment. These models are harmful self-fulfilling prophecies: their deployment harms a group of patients, but the worse outcome of these patients does not diminish the discrimination of the model. Our main result is a formal characterization of a set of such prediction models. Next, we show that models that are well calibrated before and after deployment are useless for decision-making, as they make no change in the data distribution. These results call for a reconsideration of standard practices for validation and deployment of prediction models that are used in medical decisions.

INTRODUCTION

Clinicians and medical researchers frequently employ outcome prediction models (OPMs): statistical models that predict a certain health outcome based on a patient's characteristics.[1] Researchers develop OPMs to provide information to clinicians so they may use this information in difficult treatment decisions (e.g., Salazar et al.[2]). In some cases, clinicians will treat patients with a bad expected outcome more aggressively, for example, by giving cholesterol-lowering medication to patients with a high predicted risk of a heart attack.[3,4] In other cases, for instance, when the treatment is burdensome or scarcely available (e.g., ventilator machines in the intensive care unit during a pandemic), clinicians

This is an open access article under the CC BY license (http://creativecommons.org/licenses/by/4.0/).

may reserve treatment for patients with a good predicted outcome.

Many such OPMs are added to the protocol of care by designing specific thresholds for specific actions.[3] If the predicted outcome is above or below the threshold, a certain action is taken, e.g., the patient receives a more aggressive therapy. The basis for including an OPM in a care protocol is generally predictive accuracy in validation studies.[5] In these validation studies, the OPM may or may not have been used to inform treatment decisions. While the difference between a clinical trial of an OPM's deployment and the validation of performance metrics is appreciated in the medical literature, there are still notable examples where the latter is perceived to be sufficient to justify the implementation of OPMs in the protocol of care. This is reflected in several guidelines and reviews.[5,6] Commonly used performance metrics are measures for discrimination and calibration, the latter being assessed much less frequently than the former.[7]

At first, it may seem that using OPMs for decision support is beneficial since giving more information should lead to better treatment decisions. However, implementing a prediction model for treatment decisions is an intervention that changes treatment decisions and, thus, patient outcomes. Whether this change in treatment policy improves patient outcomes is not determined by prediction accuracy in a validation study.[8] For instance, in cases where a certain patient subpopulation historically received suboptimal care, an accurate OPM will predict a worse outcome for these patients compared to similar patients from a different subpopulation. If clinicians decide to withhold effective treatments (e.g., due to scarcity or perceived futility) to this underserved subpopulation based on the OPM's prediction of a bad outcome, then the implementation of the OPM perpetuates biases and causes harm to these patients despite its accuracy. Moreover, the implementation of this harmful new policy brought about the scenario predicted by the OPM, as in a self-fulfilling prophecy. One concrete example where clinicians treat patients with a bad expected outcome less aggressively is in small cell lung cancer. Prognostic scores for patients with small cell lung cancer, such as the Manchester score,[9] are specifically intended to not overtreat patients with a bad predicted outcome because this is expected to be futile.[10,11]

Recognizing that prediction model performance may change over time, across healthcare settings, and in certain patient subgroups, many call for increased monitoring of AI models, with model updating mentioned as the best approach.[12–14] However, these approaches fall short, as they put the wrong metric upfront: prediction accuracy. We show that the value of a prediction model is not directly derived from its accuracy, and in some cases, having worse prediction accuracy over time is exactly what we want from a patient outcomes perspective. Focusing only on predictive performance might lead to the employment of a new policy that is harmful to patients or to the unduly withdrawal of a policy that was, in fact, beneficial.

In this article, we address the following questions. (1) Under what conditions is a new policy based on an OPM going to be harmful, meaning that it leads to worse outcomes than before using the model? (2) In what circumstances would such a harmful policy go undetected by measures of discrimination or calibration? In what follows, we provide a formalization of the case where patients with a high predicted probability of the outcome get treatment, where the outcome may be preferable (e.g., 1-year survival) or undesirable (e.g., a heart attack). Specifically, we examine the setting where a new OPM is supposed to "personalize" an existing treatment policy by considering additional features. The methods section provides a motivating example, notation, and definitions, and the results section presents the main results concerning OPMs that are harmful and self-fulfilling prophecies. We first show that even in a simple setup with a binary covariate, a non-trivial subset of OPMs yields harmful self-fulfilling prophecies. This means that such models cause harm but exhibit good discrimination on post-deployment data, meaning that naively interpreting this as a successful deployment leads to harmful policies. These theoretical results are paired with numerical experiments demonstrating that harmful self-fulfilling prophecies can occur without assuming extreme treatment effects or treatment effect heterogeneity. Next, perhaps surprisingly, we show that when an OPM is well calibrated on both (1) the historical data and (2) a validation study where the model is used for treatment decisions, the OPM is not useful for decision-making. Finally, after highlighting the shortcomings of validating and implementing OPMs based on predictive performance, we mention approaches to model building and validation that explicitly account for the *causal* effects of treatments on the predicted outcomes, avoiding such shortcomings.

Based on our results, several common practices in building and deploying OPMs intended for decision-making need revision. (1) Developing OPMs on observational data without regard for the historical treatment policy is potentially dangerous because the change in treatment policy between pre- and post-deployment is what determines the effect of the model on patient outcomes. (2) Implementing a personalized OPM is not always beneficial, even if the model is very accurate. (3) When monitoring discrimination prospectively after deployment, sometimes good discrimination means a harmful new policy and sometimes a beneficial one.

METHODS

Motivating example of a harmful self-fulfilling prophecy

We start with a hypothetical example based on realistic medical assumptions that would result in an OPM yielding a policy that is both harmful, meaning patient outcomes are worse compared to before deployment, and self-fulfilling, meaning the OPM has good discrimination post-deployment. In Note S1, we provide a formal version of this example with corresponding equations and proof.

Consider the problem of selecting a subset of patients with end-stage cancer for palliative radiotherapy. Such treatment has side effects, and thus, domain experts advise reducing overtreatment in this population. To comply with this advice, a medical center needs to decide which patients will not be eligible anymore for the therapy. The medical center decides to give the therapy to patients with the longest expected overall survival, under the assumption that for these patients, the side effects are justifiable. To support this policy, researchers build an OPM to predict the probability of 6-month overall survival based on the pre-treatment tumor growth rate using historical patient records from the medical center. Fast-growing tumors are more

aggressive, so these patients have a shorter survival overall. The medical center decides to implement this model to allocate radiotherapy and tests the model's discrimination post-deployment.

The new treatment policy with the OPM is, thus, "treat patients with slow-growing tumors but not those with fast-growing tumors." However, fast-growing tumors respond better to radiotherapy than slow-growing tumors,[15] so the new OPM-based policy treats exactly the wrong patients: those who do not benefit from treatment still receive it, and those who would benefit from treatment do not, so deployment of the model is harmful. The contrast in survival between patients with fast-growing tumors and slow-growing tumors is only more pronounced post-deployment, meaning that, paradoxically, the OPM has good discrimination before and after deployment.

This potential for deploying harmful self-fulfilling prophecies by only relying on measures of predictive discrimination is clearly undesirable. We now provide a formal description of when these situations occur, revealing also a dual case where OPMs that provide benefits to patient subgroups show worse post-deployment discrimination.

Notation and definitions

We assume a binary treatment T, a binary outcome Y, and a binary feature $X \in \mathcal{X} = \{0,1\}$. We denote the outcome obtained with setting treatment T to t as Y_t. An OPM is a function trained on historical data to predict the probability of the outcome of interest. We use $\pi_i(X)$ to denote a policy for assigning treatment, possibly conditional on X, with an index i to indicate what policy we are referring to. Throughout the paper, π_0 will be used to indicate the historic treatment policy that was in place in the data from which the OPM was developed.

We assume the historical policy is constant and deterministic, meaning that it is always equal to 0 or 1 (i.e., patients were always treated or never treated). Next we define what it means to craft a policy based on an existing OPM. We will be concerned only with *threshold-based policies*, namely policies that assign treatment based on a threshold $\lambda \in \mathbb{R}$. In our setup, policies assign treatment to patients only if the expected outcome is above λ, which could mean either a desirable (e.g., 1-year survival) or undesirable (e.g., a heart attack) outcome.

Definition 1 (policy informed by OPM): let $f: X \rightarrow [0,1]$ be an OPM and $\lambda \in \mathbb{R}$ a threshold. We call π_f a policy informed by f and define it as follows:

$$\pi_f(x) = \begin{cases} 1 & f(x) > \lambda \\ 0 & f(x) \leq \lambda \end{cases}. \qquad \text{(Equation 1)}$$

Such policies describe the post-deployment scenario, when the OPM influences treatment assignment. This deployment will change some of the outcome distributions compared to pre-deployment. We distinguish probabilities pre- and post-implementation using subscripts: $p_i(.)$ with $i = 0$ for the pre-implementation probabilities and $i = f$ for the post-implementation probabilities. We now present the first key idea of this paper, namely the special class of OPMs whose predictions are reinforced upon implementation. We consider as a metric of discrimination the popular area under the receiver operating characteristic curve[16] (AUC).

Definition 2 (self-fulfilling OPM): let $f: \mathcal{X} \rightarrow [0,1]$ be an OPM and $\lambda \in \mathbb{R}$ a threshold, and let π_f be the policy informed by f. Let $AUC(\pi_i)$ denote the AUC of this OPM on data generated with the historic policy (π_0) or with the policy defined by (π_f). We call the pair (f, λ) self-fulfilling if the AUC remains the same or increases post-deployment, namely iff

$$AUC(\pi_f) \geq AUC(\pi_0). \qquad \text{(Equation 2)}$$

Finally, we specify what we mean with an OPM being harmful in comparison with the status quo.

Definition 3 (harmful OPM): let $f: \mathcal{X} \rightarrow [0,1]$ be an OPM and $\lambda \in \mathbb{R}$ a threshold, let π_0 denote the historic treatment policy, and let π_f be the policy informed by f.

We write the expected outcomes under the different policies as

$$p_i(Y=1|X) = \mathbb{E}_{T \sim \pi_i(X)} p(Y_T = 1|X), \qquad \text{(Equation 3)}$$

where $i = 0$ denotes the historical distribution and $i = f$ the distribution under π_f. We call f harmful for the group with $X = x$ with $p(X = x) > 0$ if the expected outcome of this group is worse under the new policy compared to the old policy, namely when $Y = 1$ is preferable iff

$$p_f(Y=1|X=x) < p_0(Y=1|X=x) \qquad \text{(Equation 4)}$$

or when $Y = 0$ is preferable iff

$$p_f(Y=1|X=x) > p_0(Y=1|X=x). \qquad \text{(Equation 5)}$$

Note that this definition is for when deploying an OPM is harmful to a subgroup of patients, which, in general, is different from being harmful marginally, i.e., applying π_f leads to worse outcomes on average. However, we will later see that in our setup with binary X, one of the two groups has the same outcomes pre- and post-deployment, so an OPM that is harmful to a subgroup will also be marginally harmful. When a policy informed by an OPM is both harmful and self-fulfilling, we have a worst-case scenario where the new policy is causing harm to a subgroup, but this, perhaps counterintuitively, does not result in a decrease in AUC post-deployment.

RESULTS

We now move to the main results, whose proofs can be found in Note S2.

The setting where a new OPM is supposed to personalize an already existing treatment policy by considering more features is encoded as follows: the new OPM considers a feature X that was previously ignored by the historical policy, specifically, π_0 is constant and deterministic. In addition, the new policy π_f is not constant but varies with X.

Harmful models may have good discrimination post-deployment

We state our main observation as an informal theorem.

Theorem 4 (informal main result): let π_f be the policy informed by the OPM f using a threshold λ. Assume that (1) the historical

policy π_0 is constant and deterministic, (2) the new policy π_f is not constant, i.e., not always equal to 1 or 0, and (3) the marginal distribution of X is the same pre- and post-deployment: $p_i(X) = p(X)$ for $i \in \{0, f\}$.

Under these assumptions, a non-trivial subset of OPMs will demonstrate good post-deployment discrimination because they yield self-fulfilling prophecies and, at the same time, their deployment harms patients.

We proceed to characterize the contours of the subset of self-fulfilling and harmful OPMs.

Proposition 5 (self-fulfilling): suppose that the assumptions of theorem 4 hold. Furthermore, assume that the joint probabilities of X and Y are non-deterministic both pre- and post-deployment:

$$0 < p_i(Y = 1, X = x) < 1, \forall x \in \mathcal{X}. \quad \text{(Equation 6)}$$

Then, the following two statements are true: (1) if the treatment effect is always positive, namely $\forall x \in \mathcal{X} : p(Y_1 = 1|X = x) \geq p(Y_0 = 1|X = x)$, then (f, λ) is self-fulfilling, and (2) if the treatment effect is always negative, meaning $\forall x \in \mathcal{X} : p(Y_1 = 1|X = x) < p(Y_0 = 1|X = x)$, then (f, λ) is not self-fulfilling.

Proposition 5 gives sufficient conditions for an OPM to yield a self-fulfilling prophecy. When $Y = 1$ is preferable, meaning the new policy treats only those with a favorable predicted outcome (e.g., under resource scarcity), the sufficient condition is that the treatment effect is beneficial for all values of X. When instead $Y = 0$ is preferable, meaning the "treat high-risk patients" setting, the sufficient condition is that treatment is detrimental for all values of X. Treatments that are detrimental for all values of X are less likely to be used in practice, as, most often, treatments are approved for use after they are proven to be beneficial on average with a randomized controlled trial (RCT). In this case of "treat high-risk patients," self-fulfilling prophecies may still occur when the treatment is detrimental to a subgroup of patients. The assumption in proposition 5 that a treatment is always beneficial (or harmful) may hold in many cases. Typically, the *size* of the effect may vary over individuals, but that does not mean that a treatment is beneficial for patients with some values of X but harmful for patients with other values. For example, patients with a high risk of cardiovascular disease are expected to benefit more from preventative treatments, such as cholesterol-lowering medication, than patients with a low risk of cardiovascular disease, but a beneficial effect is expected in all patients. If the assumption does not hold, meaning that the treatment is beneficial for some patients and detrimental for others, then self-fulfilling prophecies may still occur, as shown later in the numerical experiments, but we can no longer provide sufficient conditions for when a self-fulfilling prophecy will occur for sure.

Remark 6: proposition 5 does not depend on the OPM's discrimination in the historical data, meaning that models with "good" discrimination (i.e., high AUC) and "bad" discrimination (low AUC) are equally susceptible to yielding self-fulfilling prophecies under the conditions of the proposition.

Now we know when OPMs are self-fulfilling and thus have good post-deployment discrimination, but can these self-fulfilling OPMs also be harmful? Proposition 7 indicates that they can.

Proposition 7 (harmful): under the assumptions of theorem 4, when $Y = 1$ is preferable, f is harmful for the group with $X = x$ iff

(1) $\pi_0(x) = 1$ and $\pi_f(x) = 0$ and $p(Y_1 = 1|X = x) > p(Y_0 = 1|X = x)$ or
(2) $\pi_0(x) = 0$ and $\pi_f(x) = 1$ and $p(Y_1 = 1|X = x) < p(Y_0 = 1|X = x)$.

When $Y = 0$ is preferable, the inequality signs reverse.

The conditions of this proposition indicate that, as one would expect, removing the treatment from the group with $X = x$ is harmful iff $p(Y_1 = 1|X = x) > p(Y_0 = 1|X = x)$ (assuming $Y = 1$ is preferable), i.e., if the effect of the treatment was positive for this group. Conversely, adding treatment to this group is damaging iff $p(Y_1 = 1|X = x) < p(Y_0 = 1|X = x)$ (when $Y = 1$ is preferable), meaning that the treatment decreases the outcome for the group.

Remark 8 (harmful OPMs are marginally harmful): under the assumptions of theorem 4, OPMs that are harmful for one subgroup are also harmful on average, as the other subgroup's treatment policy and outcomes do not change.

Taking proposition 5 on when OPMs yield self-fulfilling prophecies and proposition 7 on when OPM deployment is harmful together, we reach the perhaps surprising conclusion of theorem 4: even in the simple setup of binary treatment and binary X, some OPMs are both self-fulfilling prophecies, thus demonstrating good post-deployment discrimination, and harm a patient subgroup when deployed. We presented an example above, which we formalize in Note S1. In Table 1, we list the cases in which OPM deployment is harmful based on three pieces of information that are available post-deployment: (1) is $Y = 1$ preferable or undesirable? (2) Was the historical policy "treat everyone" or "treat no one"? (3) Did the AUC of the OPM increase post-deployment compared to the AUC pre-deployment (i.e., is the OPM self-fulfilling)? Finally, we note that the performance of the OPM on the historical data does not feature in the assumptions or statement of proposition 7. This entails, contrary to common expectations, that a high performance on historical data, including external validation, provides no guarantee of whether the OPM-driven policy will be beneficial.

In examining such results, one may wonder what would be the size of the differences in AUC for the described harmful self-fulfilling OPMs in realistic data settings and if such OPMs may also occur if the assumption of the treatment being beneficial (or harmful) for all values of X does not hold. To answer these questions, we conducted a numerical experiment via the following data distributions:

$$x \sim B(p_x), \quad \text{(Equation 7)}$$

$$t \in \{0, 1\}, \quad \text{(Equation 8)}$$

$$\eta = \beta_0 + \beta_x x + \beta_t t + \beta_{xt} xt, \text{ and} \quad \text{(Equation 9)}$$

$$y \sim B(\sigma(\eta)), \quad \text{(Equation 10)}$$

where p_x is the proportion of data points with a positive attribute X, B the Bernoulli distribution, t is the historical treatment policy (which is always 0 or 1 according to our assumptions), σ is the sigmoid (logistic) function, and the β parameters encode the effects

Table 1. Overview of when OPM deployment was harmful based on three pieces of information that were available post-deployment

Interpretation of $Y = 1$ (and policy)	π_0	$\mathrm{AUC}(\pi_f) - \mathrm{AUC}(\pi_0)$	OPM deployment was
Desirable (treat low risk patients)	1 (treat everyone)	>0 (self-fulfilling)	harmful
	1 (treat everyone)	<0 (not self-fulfilling)	beneficial
	0 (treat no one)	>0 (self-fulfilling)	beneficial
	0 (treat no one)	<0 (not self-fulfilling)	harmful
Undesirable (treat high-risk patients)	1 (treat everyone)	>0 (self-fulfilling)	beneficial
	1 (treat everyone)	<0 (not self-fulfilling)	harmful
	0 (treat no one)	>0 (self-fulfilling)	harmful
	0 (treat no one)	<0 (not self-fulfilling)	beneficial

These results apply under the assumptions of theorem 4: the historical policy π_0 is constant and deterministic, the new policy is not constant, and the marginal distribution of X (a binary variable) is the same pre- and post-deployment. As an example, the top row corresponds to the motivating example in the introduction. Before deployment, the historical policy was to treat everyone; the new policy is to treat only patients with a favorable expected outcome. Post-deployment, the OPM has better discrimination than before deployment. Because the treatment decisions (and thus outcomes) for the patients with a good prognosis have not changed, an increased AUC post-deployment can only occur when the patients with a bad prognosis have even worse outcomes than before. Thus, the deployment must have been harmful. π_0, historical treatment policy (either treat everyone or treat no one); $\mathrm{AUC}(\pi_f)$, AUC post-deployment; $\mathrm{AUC}(\pi_0)$, AUC pre-deployment; OPM, outcome prediction model.

of x and t on the outcome. Setting these parameters, enforcing the assumptions of the theorem, and deciding whether a higher Y is better (e.g., 1-year survival) or worse (e.g., a heart attack) gives enough information to describe the pre- and post-deployment scenarios. Note that a non-constant policy π_f entails that different treatments are now prescribed for the two groups defined by X; thus, further assumptions on the model and threshold λ are not needed. This allows us to calculate discrimination statistics pre- and post-deployment and to determine whether the new policy is harmful. By repeating the experiment for several values of the parameters—within reasonable ranges—one can investigate when harmful self-fulfilling policies arise.

The results match the theoretical findings and, furthermore, display that harmful self-fulfilling policies do occur in "common" circumstances without extreme treatment effects or extreme treatment effect interactions, as well as when the treatment effect is not of a constant sign. Figure 1 shows several instances of the experiment. A positive difference in AUC denotes a self-fulfilling policy, while harmful policies fall within a colored area. Inspection of the figure reveals several scenarios to be harmful and self-fulfilling in the top right and bottom left graphs. These scenarios can occur at different values of treatment effect (parameter β_t) and can even lead to an increase of AUC of >0.1. For Figure 1, we only kept settings where the treatment effect is beneficial on average. This removes several cases of harmful self-fulfilling prophecies but is more realistic, as treatments are generally only allowed on the market if their average effectiveness is demonstrated in RCTs. In Figure S3 in Note S3, all settings are presented. Furthermore, Figure S6 in Note S3 gives another visualization of the same experimental results, this time highlighting that harmful self-fulfilling prophecies occur in the absence of strong treatment effect interactions (i.e., treatment effect heterogeneity or the parameter β_{xt}).

Full details on the setup of the numerical experiment and further results can be found in Note S3, and the code to

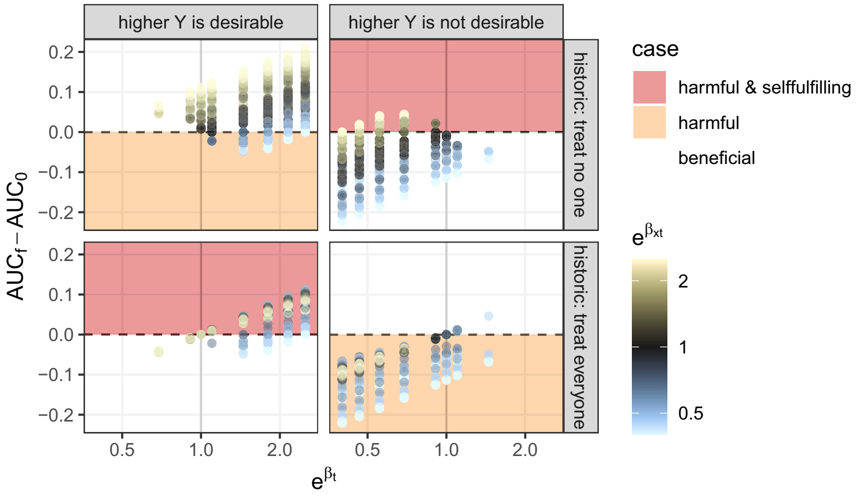

Figure 1. Results of the numerical experiment
The treatment effect is reported on the horizontal axis on the odds ratio scale (e^{β_t}), while the difference in AUC pre- and post-deployment is given on the vertical axis. When the said difference is positive, we have a self-fulfilling policy. An increase in AUC occurs when the difference in outcomes between the two groups increases post-deployment. Since our setup implies that only one group changes, this means that either the good-prognosis group got even better (a beneficial policy) or the bad-prognosis group got even worse (a harmful policy). The historic policy determines which group has a change in treatment policy and outcome. The four graphs reflect the different combinations of historical policy and outcome interpretation. Areas with (harmful) self-fulfilling prophecies are color coded. Each of the 8 areas corresponds to a row in Table 1. The points are color coded with the value of treatment effect interaction, again with an odds ratio ($e^{\beta_{tx}}$).

reproduce the results is available online (see data and code availability).

Note that the table and figures highlight a dual problem of harmful self-fulfilling, which we could call "beneficial self-defeating": the case where AUC decreases post-deployment but the new policy is, in fact, beneficial. In this case, an overreliance on performance metrics might lead to another ill-advised decision: the withdrawal of a policy that was, in fact, beneficial.

OPMs that are calibrated pre- and post-deployment are not useful for treatment decisions

Monitoring discrimination post-deployment and naively interpreting good post-deployment discrimination as a safe deployment is not a good strategy, as self-fulfilling prophecies have good post-deployment discrimination but can still be harmful depending on the context. Conversely, beneficial policies may have decreased post-deployment discrimination due to the desirable effect of improving patient outcomes.

We now turn to another key metric of OPMs predicting the risk of an outcome, calibration,[17–19] and investigate how post-deployment calibration relates to harmful policies.

We use the following definition of calibration.

Definition 9: let $p(X,Y)$ be a joint distribution over feature X and binary outcome Y and $f : \mathcal{X} \to [0,1]$ an OPM. f is calibrated with respect to $p(X,Y)$ if, for all $\alpha \in [0,1]$ in the range of f, $\mathbb{E}_{X,Y\sim p(X,Y)}[Y|f(X) = \alpha] = \alpha$.

The OPM can be calibrated on the pre-deployment historical distribution and/or the post-deployment distribution. Theorem 4 states that harmful OPMs can have good pre- and post-deployment discrimination, but can they also have good calibration?

The following theorem shows that OPMs that are calibrated pre- and post-deployment do not lead to better treatment decisions.

Theorem 10: let f be an OPM that is calibrated on historical data and π_f be non-constant. Such an OPM is calibrated on the deployment distribution iff, for every $x \in \mathcal{X}$,

$$\pi_0(x) = \pi_f(x) \text{ or } p(Y_1 = 1|X = x) = p(Y_0 = 1|X = x). \quad \text{(Equation 11)}$$

Note that this entails that for all $x \in \mathcal{X}$, either the treatment policy does not change or it changes where it is irrelevant because, for that value of X, the treatment effect is zero. Both cases imply that the implementation of the OPM is inconsequential. This may seem counterintuitive, but an OPM being calibrated both before and after deployment means the distribution has not changed, so the policy remains the same, or the policy was changed where it is irrelevant (i.e., no treatment effect). Therefore, an OPM that is calibrated on the development cohort that remains calibrated post-deployment is not a useful OPM.

Related work

The intuition that deploying models for decision support is an intervention that requires causal evaluation methods goes back to at least the 90s,[20] and previous work noted that prediction accuracy does not equal value for treatment decision-making.[8,21,22] Here, we take the additional step of exactly characterizing the set of prediction models that yield harmful self-fulfilling prophecies. The idea that model deployment changes the distribution and affects model performance was noted in several lines of previous work. Several authors noted that model performance may degrade over time due to the effect of deployment of the model,[23,24] but we study the case where model performance does *not* degrade but the implementation of it still causes harm. Also, we find that degraded discrimination may indicate a benefit of the deployment. Perdomo et al.[25] and Liley et al.[26] study the setting of performing successive model updates, each time after deploying the previous model for decision-making. Perdomo et al.[25] study when, over successive deployments, the predictive performance stabilizes or reaches optimality, and Liley et al.[26] study both model stability and the effect of model deployment on outcomes. Our work may be seen as a special case of these works with only a single model deployment and no model update, but we add new insights as we describe exactly *when* a single model deployment leads to harm and good post-deployment discrimination.

Several groups have studied out-of-distribution generalization and its connections to causality and invariance[27–29] with the aim of removing a model's dependency on spurious correlations. Again, our work differs, as we are interested in characterizing model performance following a very specific distribution change (a treatment policy change induced by a prediction model), and our main concern is the effect of this policy change on outcomes. Finally, current guidelines on prediction model validation and deployment focus on discrimination and calibration only, not on these newer invariance metrics.[5,22] Concurrent work studies the same setup as ours through the lens of domain adaptation, where each (pre-)deployment setting is formalized as a domain.[30] They describe when the effect of deploying (or updating) an OPM for decision support can be estimated without observing outcomes under the target domain; however, both their assumptions and results diverge from the present work.

We are not the first to warn against naively using OPMs for decision support (see, e.g., points 6.3 and 6.7 in Assel et al.[31]). However, (intended) misuse of OPMs is still far too common in medical research and guidelines, and the reason why this can lead to harmful situations is not well understood. Our work provides a formal approach to understanding the risks of using OPMs without proper validation.

DISCUSSION

We showed when OPMs yield harmful self-fulfilling prophecies, meaning they lead to patient harm when used for treatment decision-making but retain good discrimination after deployment. Moreover, we showed that when a model is well calibrated before and after deployment, it is not useful for treatment decision-making. The upshot of these findings is that not only do harmful and self-fulfilling policies exist, but in some scenarios, it is even *desirable* to see worse discrimination after deployment, as this may signal a beneficial new policy in terms of patient outcomes. These results demonstrate the inadequacy of evaluating predictive models post-deployment with discrimination and calibration when these models are used for decision-making.

When interpreting the performance of an OPM post-deployment, a "high AUC is good, low AUC is bad" mindset proves to be too simplistic. A higher performance post-deployment

does not necessarily indicate a beneficial policy change, and a lower performance post-deployment is not, by itself, a sign that the model is harmful. For instance, the latter may be due to poor generalization performance but may also be due to the OPM implementation being beneficial and changing the population so that the prediction task becomes harder (hence a lower AUC), shedding a new light on results such as those of Wong et al.[32] In this second circumstance, removing an OPM-based policy due to low performance would, in fact, be detrimental in terms of patient outcomes. When presented with a case where an OPM was deployed and re-evaluated for predictive accuracy post-deployment, our Table 1 can provide concrete guidance for determining whether the new policy was harmful or beneficial. In short, the pre-existing treatment policy, the interpretation of the outcome variable, and the change in AUC post-deployment can already give an indication of the effect of the new policy on patient outcomes, provided the assumptions of our settings hold.

In recent years, the United States Food and Drug Administration (FDA) and the European Medical Agency (EMA) have been developing protocols on regulating AI-based software for medical applications. The FDA's guiding principles explicitly include a total product life-cycle approach, where post-deployment monitoring and certain potential model updates are foreseen and described during initial approval, both with the aim to ensure post-deployment safety, for example, under dataset shifts but also to avoid the need for re-approval after each model update. Though their guiding principles on "good machine learning practice"[33] and "predetermined change control plans"[34] both mention post-deployment monitoring for safety, the intended monitoring seems to center mostly around predictive performance, which our results demonstrate to be insufficient to protect against harmful self-fulfilling prophecies. The EMA's "Reflection paper on the use of artificial intelligence in the life cycle of medicines" also recommends pre-planned monitoring but only of predictive performance.[35]

Requiring explicit monitoring of changes in patient outcomes over time and changes in treatment policy may, in some cases, be warranted. Though monitoring patient outcomes in important pre-determined patient subgroups before and after deployment may detect harmful model deployments, before-after comparisons are plagued by well-known biases such as potential concurrent changes in other policies or general time trends in outcomes. The best experiment to demonstrate the safety of deploying an OPM is to conduct a cluster RCT, where some caregivers are randomly selected to have access to the OPM and others are not. The difference in average outcomes of patients between the caregivers with and without access determines whether using the OPM led to better patient outcomes. When cluster randomized trials are unfeasible, other, smaller clinical studies might be the next best option.[36,37] How to pre-specify safe model monitoring and updates after deployment in a total product life-cycle approach is left for future work.

Finally, we note that developing OPMs that ignore the historic treatment policy is, in many cases, a bad approach when the ultimate aim is to improve the policy for assigning treatments.[8,38] Instead, researchers should consider using methods developed for improving decisions, such as prediction-under-intervention models or models of the conditional average treatment effect (CATE) (for example, Feuerriegel et al.,[39] Wager and Athey,[40] and Kreif et al.[41]). These methods require either the availability of large, good-quality RCTs with detailed information on pre-treatment patient characteristics or observational datasets where the assumption of no unobserved confounding is tenable. Also, these methods require a specific evaluation strategy, as they are evaluated on their ability to predict outcomes under a new treatment policy or the effect of introducing a new treatment policy on outcomes, not on their ability to predict the outcome under the historical treatment policy. For these models, cluster randomized trials are also the gold standard for evaluating the effect of the new treatment policy on patient outcomes, but other, specialized evaluation methods exist.[37,42] In this light, evaluating an OPM-based policy post-deployment with Table 1 is not a recommended practice for new OPMs but rather a way to determine post hoc whether potential harm was done when an OPM was deployed for decision support without the proper prior evaluation.

Some limitations remain, encoded in the assumptions of our formal results. The setting we describe is kept simple on purpose, a choice that helps to pinpoint the problem but somewhat limits the applicability of this theory to real-world use cases. The extension of our results to other feature types (continuous and categorical X), non-threshold-based policies, or a π_0 that is not constant (i.e., varies with X) or is non-deterministic is left to future work. Other, more complex use cases worth investigating might display policies that are harmful to subgroups identified by variables not included in the list of predictors of the model. The continuation of this line of work entails the re-evaluation of the metrics to monitor and assess a model's effectiveness, and given that model deployments for decision support are interventions, this will benefit from using the language of causal inference.

Conclusion

OPMs can yield harmful self-fulfilling prophecies when used for decision-making. The current paradigm on prediction model development, deployment, and monitoring needs to shift its primary focus away from predictive performance and instead toward changes in treatment policy and patient outcomes.

RESOURCE AVAILABILITY

Lead contact

Requests for further information and resources should be directed to and will be fulfilled by the lead contact, Wouter A.C. van Amsterdam, (w.a.c.vanamsterdam-3@umcutrecht.nl).

Materials availability

This study did not generate new unique reagents.

Data and code availability

- No data were used in this study except those generated in the numerical experiments.
- The code to reproduce the numerical experiments is deposited at Zenodo.[43]
- Any additional information required to reproduce the results reported in this paper is available from the lead contact upon request.

AUTHOR CONTRIBUTIONS

Study conception and design, W.A.C.v.A. and G.C.; writing – original draft, W.A.C.v.A. and G.C.; writing – review & editing, all authors.

DECLARATION OF INTERESTS

The authors declare no competing interests.

DECLARATION OF GENERATIVE AI AND AI-ASSISTED TECHNOLOGIES IN THE WRITING PROCESS

During the preparation of this work, the authors used ChatGPT in order to shorten the summary and the bigger picture section. After using this tool, the authors reviewed and edited the content as needed and take full responsibility for the content of the publication.

SUPPLEMENTAL INFORMATION

Supplemental information can be found online at https://doi.org/10.1016/j.patter.2025.101229.

Received: October 18, 2024
Revised: December 10, 2024
Accepted: March 12, 2025
Published: April 11, 2025

REFERENCES

1. Steyerberg, E.W. (2009). Applications of Prediction Models (Springer).
2. Salazar, R., Roepman, P., Capella, G., Moreno, V., Simon, I., Dreezen, C., Lopez-Doriga, A., Santos, C., Marijnen, C., Westerga, J., et al. (2011). Gene Expression Signature to Improve Prognosis Prediction of Stage II and III Colorectal Cancer. J. Clin. Oncol. *29*, 17–24.
3. Arnett, D.K., Blumenthal, R.S., Albert, M.A., Buroker, A.B., Goldberger, Z.D., Hahn, E.J., Himmelfarb, C.D., Khera, A., Lloyd-Jones, D., McEvoy, J.W., et al. (2019). 2019 ACC/AHA Guideline on the Primary Prevention of Cardiovascular Disease: A Report of the American College of Cardiology/American Heart Association Task Force on Clinical Practice Guidelines. Circulation *140*, e596–e646. https://doi.org/10.1161/CIR.0000000000000678.
4. Karmali, K.N., Lloyd-Jones, D.M., van der Leeuw, J., Goff, D.C., Yusuf, S., Zanchetti, A., Glasziou, P., Jackson, R., Woodward, M., Rodgers, A., et al. (2018). Blood pressure-lowering treatment strategies based on cardiovascular risk versus blood pressure: A meta-analysis of individual participant data. PLoS Med. *15*, e1002538. https://doi.org/10.1371/journal.pmed.1002538.
5. Kattan, M.W., Hess, K.R., Amin, M.B., Lu, Y., Moons, K.G.M., Gershenwald, J.E., Gimotty, P.A., Guinney, J.H., Halabi, S., Lazar, A.J., et al. (2016). American Joint Committee on Cancer acceptance criteria for inclusion of risk models for individualized prognosis in the practice of precision medicine. CA Cancer J. Clin. *66*, 370–374. https://doi.org/10.3322/caac.21339.
6. Rahimi, K., Bennett, D., Conrad, N., Williams, T.M., Basu, J., Dwight, J., Woodward, M., Patel, A., McMurray, J., and MacMahon, S. (2014). Risk prediction in patients with heart failure: a systematic review and analysis. JACC. Heart Fail. *2*, 440–446.
7. Van Calster, B., McLernon, D.J., van Smeden, M., Wynants, L., Steyerberg, E.W., Topic Group 'Evaluating diagnostic tests and prediction models' of the STRATOS initiative, Collins, G.S., Macaskill, P., McLernon, D.J., and Moons, K.G.M. (2019). Calibration: the Achilles heel of predictive analytics. BMC Med. *17*, 230. https://doi.org/10.1186/s12916-019-1466-7.
8. van Amsterdam, W.A.C., de Jong, P.A., Verhoeff, J.J.C., Leiner, T., and Ranganath, R. (2024). From algorithms to action: Improving patient care requires causality. BMC Med. Inf. Decis. Making *24*, 111. https://doi.org/10.1186/s12911-024-02513-3.
9. Cerny, T., Blair, V., Anderson, H., Bramwell, V., and Thatcher, N. (1987). Pretreatment prognostic factors and scoring system in 407 small-cell lung cancer patients. Int. J. Cancer *39*, 146–149. https://doi.org/10.1002/ijc.2910390204.
10. Hagmann, R., Zippelius, A., and Rothschild, S.I. (2022). Validation of Pretreatment Prognostic Factors and Prognostic Staging Systems for Small Cell Lung Cancer in a Real-World Data Set. Cancers *14*, 2625. https://doi.org/10.3390/cancers14112625.
11. Ferraldeschi, R., Baka, S., Jyoti, B., Faivre-Finn, C., Thatcher, N., and Lorigan, P. (2007). Modern Management of Small-Cell Lung Cancer. Drugs *67*, 2135–2152. https://doi.org/10.2165/00003495-200767150-00003.
12. Shah, N.H., Halamka, J.D., Saria, S., Pencina, M., Tazbaz, T., Tripathi, M., Callahan, A., Hildahl, H., and Anderson, B. (2024). A Nationwide Network of Health AI Assurance Laboratories. JAMA *331*, 245–249. https://doi.org/10.1001/jama.2023.26930.
13. Celi, L.A., Cellini, J., Charpignon, M.-L., Dee, E.C., Dernoncourt, F., Eber, R., Mitchell, W.G., Moukheiber, L., Schirmer, J., Situ, J., et al. (2022). Sources of bias in artificial intelligence that perpetuate healthcare disparities—A global review. PLOS Digit. Health *1*, e0000022. https://doi.org/10.1371/journal.pdig.0000022.
14. Futoma, J., Simons, M., Panch, T., Doshi-Velez, F., and Celi, L.A. (2020). The myth of generalisability in clinical research and machine learning in health care. Lancet. Digit. Health *2*, e489–e492. https://doi.org/10.1016/S2589-7500(20)30186-2.
15. Breur, K. (1966). Growth rate and radiosensitivity of human tumours—II: Radiosensitivity of human tumours. Eur. J. Cancer *2*, 173–188. https://doi.org/10.1016/0014-2964(66)90009-0.
16. Hanley, J.A., and McNeil, B.J. (1982). The meaning and use of the area under a receiver operating characteristic (ROC) curve. Radiology *143*, 29–36. https://doi.org/10.1148/radiology.143.1.7063747.
17. Alba, A.C., Agoritsas, T., Walsh, M., Hanna, S., Iorio, A., Devereaux, P.J., McGinn, T., and Guyatt, G. (2017). Discrimination and calibration of clinical prediction models: users' guides to the medical literature. JAMA *318*, 1377–1384.
18. Huang, Y., Li, W., Macheret, F., Gabriel, R.A., and Ohno-Machado, L. (2020). A tutorial on calibration measurements and calibration models for clinical prediction models. J. Am. Med. Inf. Assoc. *27*, 621–633.
19. Van Calster, B., McLernon, D.J., Van Smeden, M., Wynants, L., and Steyerberg, E.W.; Topic Group 'Evaluating diagnostic tests and prediction models' of the STRATOS initiative (2019). Calibration: the Achilles heel of predictive analytics. BMC Med. *17*, 230–237.
20. Cooper, G.F., Aliferis, C.F., Ambrosino, R., Aronis, J., Buchanan, B.G., Caruana, R., Fine, M.J., Glymour, C., Gordon, G., Hanusa, B.H., et al. (1997). An evaluation of machine-learning methods for predicting pneumonia mortality. Artif. Intell. Med. *9*, 107–138.
21. Vickers, A.J., and Elkin, E.B. (2006). Decision curve analysis: a novel method for evaluating prediction models. Med. Decis. Mak. *26*, 565–574.
22. Moons, K.G.M., Altman, D.G., Reitsma, J.B., Ioannidis, J.P.A., Macaskill, P., Steyerberg, E.W., Vickers, A.J., Ransohoff, D.F., and Collins, G.S. (2015). Transparent Reporting of a multivariable prediction model for Individual Prognosis Or Diagnosis (TRIPOD): Explanation and Elaboration. Ann. Intern. Med. *162*, W1.
23. Lenert, M.C., Matheny, M.E., and Walsh, C.G. (2019). Prognostic models will be victims of their own success, unless…. J. Am. Med. Inf. Assoc. *26*, 1645–1650. https://doi.org/10.1093/jamia/ocz145.
24. Sperrin, M., Jenkins, D., Martin, G.P., and Peek, N. (2019). Explicit causal reasoning is needed to prevent prognostic models being victims of their own success. J. Am. Med. Inf. Assoc. *26*, 1675–1676. https://doi.org/10.1093/jamia/ocz197.
25. Perdomo, J., Zrnic, T., Mendler-Dünner, C., and Hardt, M. (2020). Performative Prediction. In Proceedings of the 37th International Conference on Machine Learning (PMLR), pp. 7599–7609.

CellPress
OPEN ACCESS

26. Liley, J., Emerson, S., Mateen, B., Vallejos, C., Aslett, L., and Vollmer, S. (2021). Model updating after interventions paradoxically introduces bias. In Proceedings of the 24th International Conference on Artificial Intelligence and Statistics (PMLR), pp. 3916–3924.

27. Arjovsky, M., Bottou, L., Gulrajani, I., and Lopez-Paz, D. (2020). Invariant Risk Minimization. Preprint at arXiv. https://doi.org/10.48550/arXiv.1907.02893.

28. Wald, Y., Feder, A., Greenfeld, D., and Shalit, U. (2021). On Calibration and Out-of-Domain Generalization. In Advances in Neural Information Processing Systems (Curran Associates, Inc.), pp. 2215–2227.

29. Puli, A.M., Zhang, L.H., Oermann, E.K., and Ranganath, R. (2022). Out-of-distribution Generalization in the Presence of Nuisance-Induced Spurious Correlations. In The Tenth International Conference on Learning Representations, ICLR 2022 (ICLR).

30. Boeken, P., Zoeter, O., and Mooij, J. (2024). Evaluating and Correcting Performative Effects of Decision Support Systems via Causal Domain Shift. In Proceedings of the Third Conference on Causal Learning and Reasoning (PMLR), pp. 551–569.

31. Assel, M., Sjoberg, D., Elders, A., Wang, X., Huo, D., Botchway, A., Delfino, K., Fan, Y., Zhao, Z., Koyama, T., et al. (2019). Guidelines for Reporting of Statistics for Clinical Research in Urology. Eur. Urol. *75*, 358–367. https://doi.org/10.1016/j.eururo.2018.12.014.

32. Wong, A., Otles, E., Donnelly, J.P., Krumm, A., McCullough, J., DeTroyerCooley, O., Pestrue, J., Phillips, M., Konye, J., Penoza, C., et al. (2021). External validation of a widely implemented proprietary sepsis prediction model in hospitalized patients. JAMA Intern. Med. *181*, 1065–1070.

33. FDA (2021). Good Machine Learning Practice for Medical Device Development: Guiding Principles. https://www.fda.gov/medical-devices/software-medical-device-samd/good-machine-learning-practice-medical-device-development-guiding-principles.

34. FDA (2023). Predetermined Change Control Plans for Machine Learning Enabled Medical Devices: Guiding Principles. https://www.fda.gov/medical-devices/software-medical-device-samd/predetermined-change-control-plans-machine-learning-enabled-medical-devices-guiding-principles.

35. EMA (2023). EMA Reflection paper on the use of artificial intelligence in the lifecycle of medicines. https://www.ema.europa.eu/en/news/reflection-paper-use-artificial-intelligence-lifecycle-medicines.

36. Vasey, B., Nagendran, M., Campbell, B., Clifton, D.A., Collins, G.S., Denaxas, S., Denniston, A.K., Faes, L., Geerts, B., Ibrahim, M., et al. (2022). Reporting guideline for the early stage clinical evaluation of decision support systems driven by artificial intelligence: DECIDE-AI. BMJ *377*, e070904.

37. Janse, R.J., Stel, V.S., Jager, K.J., Tripepi, G., Zoccali, C., Dekker, F.W., and van Diepen, M. (2024). When impact trials are not feasible: Alternatives to study the impact of prediction models on clinical practice. Nephrol. Dial. Transplant. *40*, 27–33. https://doi.org/10.1093/ndt/gfae170.

38. van Geloven, N., Keogh, R.H., van Amsterdam, W., Cinà, G., Krijthe, J.H., Peek, N., Luijken, K., Magliacane, S., Morzywołek, P., van Ommen, T., et al. (2024). The risks of risk assessment: Causal Blind Spots When Using Prediction Models for Treatment Decisions. Preprint at arXiv. https://doi.org/10.48550/arXiv.2402.17366.

39. Feuerriegel, S., Frauen, D., Melnychuk, V., Schweisthal, J., Hess, K., Curth, A., Bauer, S., Kilbertus, N., Kohane, I.S., and Van Der Schaar, M. (2024). Causal machine learning for predicting treatment outcomes. Nat. Med. *30*, 958–968. https://doi.org/10.1038/s41591-024-02902-1.

40. Wager, S., and Athey, S. (2018). Estimation and Inference of Heterogeneous Treatment Effects using Random Forests. J. Am. Stat. Assoc. *113*, 1228–1242. https://doi.org/10.1080/01621459.2017.1319839.

41. Kreif, N., DiazOrdaz, K., Moreno-Serra, R., Mirelman, A., Hidayat, T., and Suhrcke, M. (2022). Estimating heterogeneous policy impacts using causal machine learning: A case study of health insurance reform in Indonesia. Health Serv. Outcome Res. Methodol. *22*, 192–227. https://doi.org/10.1007/s10742-021-00259-3.

42. Keogh, R.H., and Van Geloven, N. (2024). Prediction Under Interventions: Evaluation of Counterfactual Performance Using Longitudinal Observational Data. Epidemiology *35*, 329–339. https://doi.org/10.1097/EDE.0000000000001713.

43. van Amsterdam, W. (2025). Numerical experiments for "When accurate prediction models yield harmful self-fulfilling prophecies", Patterns 2025. Zenodo. https://doi.org/10.5281/zenodo.14871768.

Patterns, Volume *6*

Supplemental information

When accurate prediction models

yield harmful self-fulfilling prophecies

Wouter A.C. van Amsterdam, Nan van Geloven, Jesse H. Krijthe, Rajesh Ranganath, and Giovanni Cinà

Supplemental Note S1 Hypothetical example of a harmful self-fulfilling prophecy

We now give a full-fledged hypothetical example based on realistic assumptions that would result in an OPM yielding a policy that is both harmful and self-fulfilling.

Consider the problem of selecting a subset of end-stage cancer patients for palliative radiotherapy. Such treatment has severe side-effects and thus domain experts advise to attempt to reduce over-treatment in the population of cancer patients. To comply with this advice, a medical center needs to decide which patients will not be eligible anymore for the therapy.

The medical center decides to give the therapy to patients with the longest expected overall survival, under the assumption that these patients would be those for whom the side-effects are justifiable. To support this policy, researchers built an OPM to predict the probability of 6-months overall survival based on pre-treatment tumor growth rate using historical patient records from the medical center. Fast-growing tumors are more aggressive so these patients have a shorter survival overall. The medical center decides to use this model to allocate the therapy and tests the model's discrimination post deployment. Based on this we have the following facts:

1. $X = 1$: fast growing tumor, $X = 0$: slow-growing tumor;
2. $\pi_0(X) = 1$, the historical policy was treating everyone;
3. $p(Y_0 = 1|X = 0) - p(Y_0 = 1|X = 1) > 0$, with radiotherapy, patients with fast growing tumors live shorter

A model with a good fit to the data will predict that patients with slow-growing tumors have a higher probability of 6-months survival. We also assume that the new policy is non-constant and favors those with highest predicted outcome, which means that the new policy will be 'treat patients with slow growing tumors but not those with fast growing tumors':

$$\pi_f(X) = 1 - X$$

However, it is well known that fast-growing tumors respond better to radiotherapy than slow growing tumors[15]. Based on this we add the following two assumptions:

1. $p(Y_0 = 1|X = 0) - p(Y_1 = 1|X = 0) = 0$, radiotherapy is not effective against slow growing tumors;
2. $\delta := p(Y_0 = 1|X = 1) - p(Y_1 = 1|X = 1) < 0$, radiotherapy *is* effective for fast growing tumors.

This means that the antecedent of Proposition 5 is satisfied, meaning that f yields a self-fulfilling prophecy in combination with any threshold λ such that the resulting policy is non-constant. Removing the therapy from the group $X = 1$ will worsen their outcomes by δ, separating the two groups even more and resulting in higher AUC post-deployment.

Moreover, according to the first case of Proposition 7, the OPM is harmful because the new treatment policy leads to worse outcomes for the group with fast growing tumors ($X = 1$). So the OPM-based policy treats exactly the wrong patients: those who do not benefit from treatment still receive it, those who would benefit from treatment do not, but paradoxically it has good discrimination before and after deployment.

Supplemental Note S2 Proofs of main results

Proof of Proposition 5.

Proof

First we give some elementary definitions and equalities. Define

$$\mu_i(x) = p_i(Y=1|X=x) = (1-\pi_i(x))\,p(Y_0=1|X=x) + \pi_i(x)p(Y_1=1|X=x) \tag{12}$$

So by the law of total probability we can write

$$p_i(Y=1) = p_i(X=0)\mu_i(0) + p_i(X=1)\mu_i(1) \tag{13}$$

By Bayes rule we have:

$$p_i(X=x|Y=y) = \frac{p_i(Y=y|X=x)p(X=x)}{p_i(Y=y)} \tag{14}$$

Filling in the definition of $\mu_i(x)$ into 14 using the assumption that $p_i(X=x) = p(X=x)$ we have in particular:

$$p_i(X=x|Y=1) = \frac{\mu_i(x)p(X=x)}{p_i(Y=1)} \tag{15}$$

ROC-curves are created by transforming a continuous-valued function to a binary prediction based on a varying *threshold* τ and calculating the *sensitivity* and *specificity* for each value of τ:

$$\text{sensitivity} = p(f(X) \geq \tau|Y=1) \tag{16}$$

$$\text{specificity} = p(f(X) < \tau|Y=0) \tag{17}$$

For each possible threshold, all predictions under the threshold are labeled *negative* and all predictions greater or equal to the threshold *positive*. In the case of a binary X, $f(X)$ only takes two unique values so the ROC-curve is given by just three points:

1. sensitivity $=1$, specificity $=0$ ($\tau = -\infty$)
2. sensitivity $=0$, specificity $=1$ ($\tau = +\infty$)
3. sensitivity $=$ sens, specificity $=$ spec ($\tau = \max_X f(X)$)

See Figure S1. We can directly calculate the AUC by dividing the area under the ROC-curve in two adjacent non-overlapping triangles. This gives us the following expression for the AUC (see also[43]):

$$\text{AUC} = \frac{1}{2}\text{sens} + \frac{1}{2}\text{spec} \tag{18}$$

In this binary case, the area-under the ROC curve is thus determined by a single point denoted as (spec,sens). A pair (f, λ) is self-fulfilling when:

$$\text{AUC}(f) - \text{AUC}(0) = \frac{1}{2}\left(\text{sens}_f + \text{spec}_f - \text{sens}_0 - \text{spec}_0\right) \geq 0 \tag{19}$$

We structure the proof by first creating an enumeration over all possible scenarios. We assumed π_f is non-constant, which implies that f varies with X. Since X is binary, it must be that either $f(0) > f(1)$ or $f(1) > f(0)$. These cases are symmetric under relabeling of X so without loss of generality we proceed assuming that $f(0) > f(1)$ is the case. Since π_f is not constant but π_0 is, it must be that either the treatment policy changes for $X=0$ but remains the same for $X=1$, or vice versa. This in turn implies that either $\mu_f(0) = \mu_0(0)$ or $\mu_f(1) = \mu_0(1)$.

To provide a proof for the theorem, we enumerate all the subcases based on two factors:

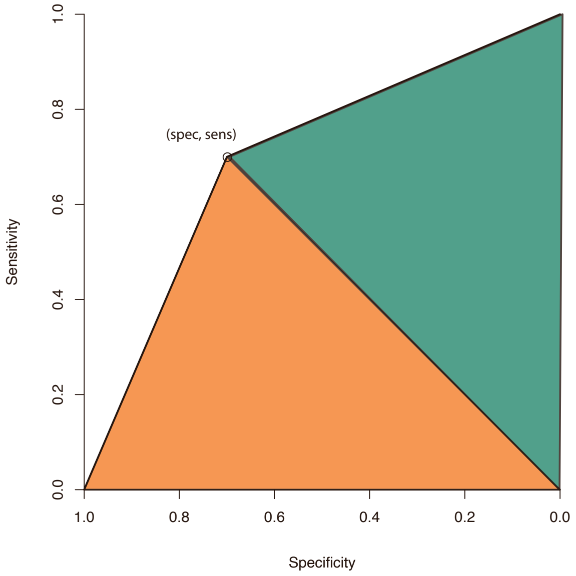

Figure S1: AUC for a binary predictor X

1. for which group does the policy change ($X = 0$ or $X = 1$)?
2. for the group with the policy change, does the outcome under the new policy remain the same (the policy is inconsequential as the treatment effect is zero), increase or decrease (this will be beneficial or detrimental depending on whether $Y = 1$ is good or bad)

This leads to the following 6 cases:

- policy change for which X?
  0. $\pi_f(0) \neq \pi_0(0)$
     effect of policy change:
     =: $\mu_f(0) = \mu_0(0)$, $\mu_f(1) = \mu_0(1)$
     <: $\mu_f(0) < \mu_0(0)$, $\mu_f(1) = \mu_0(1)$
     >: $\mu_f(0) > \mu_0(0)$, $\mu_f(1) = \mu_0(1)$
  1. $\pi_f(1) \neq \pi_0(1)$
     effect of policy change:
     =: $\mu_f(0) = \mu_0(0)$, $\mu_f(1) = \mu_0(1)$
     <: $\mu_f(0) = \mu_0(0)$, $\mu_f(1) < \mu_0(1)$
     >: $\mu_f(0) = \mu_0(0)$, $\mu_f(1) > \mu_0(1)$

These 6 combinations cover all possibilities. Since we have that $f(0) > f(1)$, by assumption of a non-deterministic $\pi_f(x) = I_{f(x)>\lambda}$ it must be that for all subcases $\pi_f(0) = 1$ and $\pi_f(1) = 0$. Each of these cases have implications for π_0 and, depending on which policy changes, $p(Y_1 = 1|X = 0) - p(Y_0 = 1|X = 0)$ or $p(Y_1 = 1|X = 1) - p(Y_0 = 1|X = 1)$. For instance case $(0, >)$ specifies that $\pi_f(0) \neq \pi_0(0)$. Because $\pi_f(0) = 1$, it follows that $\pi_0 = 0$. And because $Y_1(0) = \mu_f(0) > \mu_0(0) = Y_0(0)$ it must be that $p(Y_1 = 1|X = 0) - p(Y_0 = 1|X = 0) > 0$, meaning that the treatment increases the outcome for the group with $X = 0$.

In the two cases where the outcomes do not change ($(0, =)$ and $(1, =)$), (f, λ) is trivially self-fulfilling as nothing changes in the distribution of X, Y so the sensitivity and specificity remain the same.

We first prove self-fulfillingness in cases $(0, >)$ and $(0, <)$:

Case $(0,>)$ **and** $(0,<)$ We first address case $(0,>)$, which gives us this information:

- $\pi_f(0) \neq \pi_0(0)$
- $\mu_f(0) > \mu_0(0)$
- $\mu_f(1) = \mu_0(1)$

Since $f(0) > f(1)$ we get these sensitivity and specificity:

$$\text{sens}_i = p_i(f(X) \geq \max(f)|Y=1) = p_i(X=0|Y=1) \tag{20}$$
$$\text{spec}_i = p_i(f(X) < \max(f)|Y=0) = p_i(X=1|Y=0) \tag{21}$$

with $i \in \{0, f\}$. Plugging this into 19 yields:

$$\begin{aligned}\text{AUC}(f) - \text{AUC}(0) &= \frac{1}{2}\big(p_f(X=0|Y=1) - p_0(X=0|Y=1) \\ &\quad + p_f(X=1|Y=0) - p_0(X=0|Y=0)\big) \\ &= \frac{1}{2}\big(\mu_f(0)\frac{p(X=0)}{p_f(Y=1)} - \mu_0(0)\frac{p(X=0)}{p_0(Y=1)} \\ &\quad + (1-\mu_f(1))\frac{p(X=1)}{p_f(Y=0)} - (1-\mu_0(1))\frac{p(X=1)}{p_0(Y=0)}\big)\end{aligned}$$

where the first equality is by substitution and rearrangement, and the second by Bayes rule. We can determine the sign of this difference based on the sign of two terms:

$$= \frac{1}{2}\big(p(X=0)\big(\frac{\mu_f(0)}{p_f(Y=1)} - \frac{\mu_0(0)}{p_0(Y=1)}\big) \tag{22}$$
$$+ p(X=1)\big(\frac{1-\mu_f(1)}{p_f(Y=0)} - \frac{1-\mu_0(1)}{p_0(Y=0)}\big)\big) \tag{23}$$

We write the difference between pre- and post-deployment expected outcome for the group $X=0$ as

$$\delta := \mu_f(0) - \mu_0(0) \tag{24}$$

This gives us

$$p_f(Y=1) = p(X=1)\mu_f(1) + p(X=0)\mu_f(0) \tag{25}$$
$$= p(X=1)\mu_0(1) + p(X=0)(\mu_0(0) + \delta) \tag{26}$$
$$= p_0(Y=1) + p(X=0)\delta \tag{27}$$

where the first step is the law of total probability, the second by the definition of δ and the case information $\mu_f(1) = \mu_0(1)$, and finally again using the law of total probability. Furthermore

$$p_f(Y=0) = 1 - p_f(Y=1) \tag{28}$$
$$= 1 - p_0(Y=1) - p(X=0)\delta \tag{29}$$
$$= p_0(Y=0) - p(X=0)\delta \tag{30}$$

where the second step is by our previous calculation and the other two just the property of binary outcomes. We can now determine the signs of the two terms in 22.

$$\text{sign}\big[\frac{\mu_f(0)}{p_f(Y=1)} - \frac{\mu_0(0)}{p_0(Y=1)}\big] = \text{sign}\big[\frac{\mu_f(0)p_0(Y=1) - \mu_0(0)p_f(Y=1)}{p_f(Y=1)p_0(Y=1)}\big] \tag{31}$$

$$= \text{sign}\big[\mu_f(0)p_0(Y=1) - \mu_0(0)p_f(Y=1)\big] \tag{32}$$

The first equality is cross-multiplying, the second equality is because the product of two probabilities (which are positive by assumption) is always a positive number.

Filling in the definition of δ :

$$\text{sign}\big[\frac{\mu_f(0)}{p_f(Y=1)} - \frac{\mu_0(0)}{p_0(Y=1)}\big] \tag{33}$$

$$= \text{sign}\big[(\mu_0(0)+\delta)p_0(Y=1) - \mu_0(0)(p_0(Y=1) + p(X=0)\delta)\big] \tag{34}$$

$$= \text{sign}\big[\delta p_0(Y=1) - \mu_0(0)p(X=0)\delta\big] \tag{35}$$

$$= \text{sign}\big[\delta(p_0(Y=1) - \mu_0(0)p(X=0))\big] \tag{36}$$

$$= \text{sign}\big[\delta\mu_0(1)p(X=1)\big] \tag{37}$$

$$= \text{sign}\big[\delta\big] \tag{38}$$

In the second equality we remove canceling terms. In the third equality we pull out δ. In the fourth equality we use the expansion of $p_0(Y=1) = p(X=0)\mu_0(0) + p(X=1)\mu_0(1)$, and for the final equation we note again that $\mu_0(1)$ and $p(X=1)$ are positive probabilities so the sign is determined by the sign of δ.

Now for the second term of 22:

$$\text{sign}\big[\frac{1-\mu_f(1)}{p_f(Y=0)} - \frac{1-\mu_0(1)}{p_0(Y=0)}\big] = \text{sign}\big[\frac{1-\mu_0(1)}{p_f(Y=0)} - \frac{1-\mu_0(1)}{p_0(Y=0)}\big] \tag{39}$$

$$= \text{sign}\big[\big(1-\mu_0(1)\big)\big(\frac{1}{p_f(Y=0)} - \frac{1}{p_0(Y=0)}\big)\big] \tag{40}$$

$$= \text{sign}\big[\frac{1}{p_f(Y=0)} - \frac{1}{p_0(Y=0)}\big] \tag{41}$$

$$= \text{sign}\big[\frac{p_0(Y=0) - p_f(Y=0)}{p_f(Y=0)p_0(Y=0)}\big] \tag{42}$$

$$= \text{sign}\big[p_0(Y=0) - p_f(Y=0)\big] \tag{43}$$

$$= \text{sign}\big[p_0(Y=0) - p_0(Y=0) + p(X=0)\delta\big] \tag{44}$$

$$= \text{sign}\big[p(X=0)\delta\big] \tag{45}$$

$$= \text{sign}\big[\delta\big] \tag{46}$$

The first equality uses the case assumption that $\mu_f(1) = \mu_0(1)$. The second equality pulls out the common term $(1-\mu_0(1))$. The third equality follows because $0 < \mu_0(1) < 1$. The fourth and fifth equality are cross-multiplying and again using the positive probability property. In the sixth equality we substitute in the definition of δ. The seventh equality removes the canceling terms, and the final equality again relies on that $0 < p(X=0)$.

So both terms in 22 have the sign of δ. In subcase $(0,>)$ δ has positive sign, so

$$\text{AUC}(f) - \text{AUC}(0) > 0$$

and (f,λ) is self-fulfilling.

Immediately it is clear that in subcase $(0,<)$, (f,λ) is not self-fulfilling, as subcase $(0,<)$ equals subcase $(0,>)$ in all respects except that instead it has a negative sign for δ.

Case $(1, >)$ **and** $(1, <)$ We first address case $(1, >)$, which gives us this information:

- $\pi_f(1) \neq \pi_0(1)$
- $\mu_f(0) = \mu_0(0)$
- $\mu_f(1) > \mu_0(1)$

Again we write the difference between pre- and post-deployment expected outcome as δ, this time for the group $X = 1$:

$$\delta := \mu_f(1) - \mu_0(1) \tag{47}$$

This gives us

$$\begin{aligned} p_f(Y = 1) &= p(X = 1)\mu_f(1) + p(X = 0)\mu_f(0) && (48)\\ &= p(X = 1)(\mu_0(1) + \delta) + p(X = 0)\mu_0(0) && (49)\\ &= p_0(Y = 1) + p(X = 1)\delta && (50) \end{aligned}$$

where the first step is the law of total probability, the second by the definition of δ and the case information $\mu_f(0) = \mu_0(0)$, and finally again using the law of total probability. Furthermore

$$\begin{aligned} p_f(Y = 0) &= 1 - p_f(Y = 1) && (51)\\ &= 1 - p_0(Y = 1) - p(X = 1)\delta && (52)\\ &= p_0(Y = 0) - p(X = 1)\delta && (53) \end{aligned}$$

where the second step is by our previous calculation and the other two just the property of binary outcomes. We can now determine the signs of the two terms in 22.

The first two steps for the first are the same as in the case $(0, >)$ (see Equation 31), after these steps we substitute in the new definition of δ:

$$\begin{aligned} &\text{sign}\Big[\frac{\mu_f(0)}{p_f(Y = 1)} - \frac{\mu_0(0)}{p_0(Y = 1)}\Big] && (54)\\ &= \text{sign}\big[\mu_f(0)p_0(Y = 1) - \mu_0(0)p_f(Y = 1)\big] && (55)\\ &= \text{sign}\big[\mu_0(0)p_0(Y = 1) - \mu_0(0)(p_0(Y = 1) + p(X = 1)\delta)\big] && (56)\\ &= \text{sign}\big[-\mu_0(0)p(X = 0)\delta\big] && (57)\\ &= \text{sign}\big[-\delta\big] && (58) \end{aligned}$$

In the third equality we remove canceling terms. For the final equation we note again that $\mu_0(0)$ and $p(X = 0)$ are positive probabilities so the sign is determined by the sign of δ.

Now for the second term of 22:

subcase		π_0	$\pi_f(0)$	$\pi_f(1)$	CATE(0)	CATE(1)	self-fulfilling
0	=	0	1	0	0		yes
0	<	0	1	0	-		no
0	>	0	1	0	+		yes
1	=	1	1	0		0	yes
1	<	1	1	0		+	yes
1	>	1	1	0		-	no

Table S1: Enumeration of all possible subcases. The first column indicates for which value of X the treatment policy changes. The second column indicates whether this change improves outcomes for that group ($>$), reduces outcomes ($<$) or is irrelevant ($=$). $+/-$ indicates the sign of the subgroup treatment effect $\text{CATE}(x) := p(Y_1 = 1|X = x) - p(Y_0 = 1|X = x)$;

$$\text{sign}\Big[\frac{1-\mu_f(1)}{p_f(Y=0)} - \frac{1-\mu_0(1)}{p_0(Y=0)}\Big] \tag{59}$$

$$= \text{sign}\Big[\frac{(1-\mu_f(1))p_0(Y=0) - (1-\mu_0(1))p_f(Y=0)}{p_f(Y=0)p_0(Y=0)}\Big] \tag{60}$$

$$= \text{sign}\big[(1-\mu_f(1))p_0(Y=0) - (1-\mu_0(1))p_f(Y=0)\big] \tag{61}$$

$$= \text{sign}\big[(1-(\mu_0(1)+\delta))p_0(Y=0) - (1-\mu_0(1))(p_0(Y=0) - p(X=1)\delta)\big] \tag{62}$$

$$= \text{sign}\big[-\delta p_0(Y=0) - (1-\mu_0(1))(-p(X=1)\delta)\big] \tag{63}$$

$$= \text{sign}\big[-\delta(p_0(Y=0) - (1-\mu_0(1))p(X=1))\big] \tag{64}$$

$$= \text{sign}\big[-\delta((1-\mu_0(0))p(X=0))\big] \tag{65}$$

$$= \text{sign}\big[-\delta\big] \tag{66}$$

The first equality uses cross-multiplication to gather the sum. The second equality follows because we're dividing by a positive number. The third equality is filling in the definition on δ. The fourth equality removes canceling terms. The fifth equality factors out $-\delta$. The seventh equality is by the law of total probability.

So both terms in 22 have the sign of $-\delta$. In subcase $(1, >)$ δ has positive sign, so

$$\text{AUC}(f) - \text{AUC}(0) < 0$$

and (f, λ) is not self-fulfilling.

Immediately it is clear that in subcase $(1, <)$, (f, λ) is self-fulfilling, as subcase $(1, <)$ equals subcase $(1, >)$ in all respects except that instead it has a negative sign for δ.

Enumerating all the cases As said, in the two cases where the outcomes do not change $((0, =), (1, =))$, (f, λ) is trivially self-fulfilling.

Putting all the pieces of information for all subcases together in Table S1 we see that when $p(Y_1 = 1|X = x) - p(Y_0 = 1|X = x) \geq 0$ (the treatment effect is never negative), (f, λ) is self-fulfilling. Also, when $p(Y_1 = 1|X = x) - p(Y_0 = 1|X = x) < 0$ (the treatment effect is always negative), (f, λ) is never self-fulfilling. These observations conclude the proof.

■

Proof of Proposition 7.

Given that we assumed binary T and X, we can write the expected value of the outcome conditional on these two variables with four parameters without making parametric assumptions, marginalizing over other

variables different than X and T. For ease of interpretation of our results we write the expected value as a sum:

$$p(Y_{T=t} = 1|X = x) = \alpha + \beta_x x + \beta_t t + \beta_{xt} xt \tag{67}$$

Note that this is not an assumption on the generating process of the outcome Y, which could have arbitrary form, it is only a formal device to represent the four outcomes of interest, one for each value of X and T.

We now proceed to prove the Proposition for the case where higher outcome is better; to obtain a proof for the symmetric case (higher outcome is worse) one needs only to switch the sign in the inequalities 68 and 69, along with their specialization in the subcases.

Proof A treatment is harmful for the group with $X = x'$ iff $p_f(Y = 1|X = x') < p_0(Y = 1|X = x')$, where according to definition 3 $p_i(Y = 1|X) = \mathbb{E}_{T\sim\pi_i(X)}\, p(Y_T = 1|X)$ The proof continues as a case distinction depending on the value of x'.

Case $x' = 1$**.** For $x' = 1$ the definition of harmful translates to

$$(\pi_f(1) - \pi_0(1))\,(\beta_t + \beta_{xt}) < 0 \tag{68}$$

We consider the possible values of π_f and π_0 in subcases. Note that if $\pi_f(1) = \pi_0(1)$ the above inequality cannot hold since all terms cancel out and the treatment cannot be harmful (because nothing changes for group $X = 1$), so we only consider subcases where these two differ.

Subcase 1. We have $\pi_f(1) = 0, \pi_f(0) = 1$ and $\pi_0(x) = 1$. In this scenario, we were treating everyone and with the new policy we withhold treatment from group $X = 1$. In this case statement 68 specializes to $\beta_t + \beta_{xt} > 0$, meaning that treatment was beneficial and removing it will do damage to group $X = 1$.

Subcase 2. We have $\pi_f(1) = 1, \pi_f(0) = 0$ and $\pi_0(x) = 0$. In this scenario, we were treating nobody and with the new policy we introduce treatment for group $X = 1$. In this case statement 68 specializes to $\beta_t + \beta_{xt} < 0$, meaning that treatment is harmful and adding it damages group $X = 1$.

Case $x' = 0$**.** For $x' = 0$ the definition of harmful translates to

$$(\pi_f(0) - \pi_0(0))\,\beta_t < 0 \tag{69}$$

Again if $\pi_f(0) = \pi_0(0)$ the above inequality cannot hold since all terms cancel out and the treatment cannot be harmful (because nothing changes for group $X = 0$), so we only consider subcases where these two differ.

Subcase 1. We have $\pi_f(1) = 0, \pi_f(0) = 1$ and $\pi_0(x) = 0$. In this scenario, we were treating nobody and with the new policy we introduce treatment from group $X = 0$. In this case the statement 69 specializes to $\beta_t < 0$, which is what we intended to prove.

Subcase 2. We have $\pi_f(1) = 1, \pi_f(0) = 0$ and $\pi_0(x) = 1$. In this circumstance statement 69 specializes to $\beta_t > 0$. ■

Proof of Theorem 10.

By assumption f is calibrated on the historical data, so:

$$f(X = x) = p_0(Y = 1|X = x) = \mathbb{E}_{T\sim\pi_0(x)}\, p(Y_T = 1|X = x).$$

We now prove that f is calibrated on the deployment distribution generated by π_f iff for all $x \in \mathcal{X}$:

$$\pi_0(x) = \pi_f(x) \;\; or \;\; p(Y_1 = 1|X = x) = p(Y_0 = 1|X = x) \tag{70}$$

Proof As a shorthand define:

$$\begin{aligned}\mu_i(x) \coloneqq &p_i(Y=1|X=x)\\ =&(1-\pi_i(x))p(Y_0=1|X=x)+\pi_i(x)p(Y_1=1|X=x).\end{aligned}$$

f is calibrated on the historical data so:

$$f(X=x)=\mu_0(x), \forall x \in \mathcal{X}. \tag{71}$$

By definition, f is calibrated on the post-deployment distribution when for all $\alpha \in [0,1]$ in the range of f, $\mathbb{E}_{X,Y\sim p_f(X,Y)}[Y|f(X)=\alpha]=\alpha$. So if f is calibrated on both the historic distribution and the post-deployment distribution we have that:

$$\begin{aligned}&\mathbb{E}_{X,Y\sim p_f(X,Y)}[Y|f(X)=\alpha]\\ =&\mathbb{E}_{X,Y\sim p_f(X,Y)|f(X)=\alpha}[Y]\\ =&\mathbb{E}_{X,Y\sim p_f(X,Y)}[Y1[f(X)=\alpha]]/\mathbb{E}_{X\sim p_f(X)}[1[f(X)=\alpha]]\\ =&\mathbb{E}_{X,Y\sim p_0(X,Y)}[Y1[f(X)=\alpha]]/\mathbb{E}_{X\sim p_0(X)}[1[f(X)=\alpha]]\end{aligned}$$

Where 1[..] is used for the indicator function. We first show that this holds iff for every $x \in \mathcal{X}$, $f(x)=\mu_0(x)=\mu_f(x)$. Note that in the last two equations above, the denominators are the same as $p_0(X)=p_f(X)$, so also the enumerators must be the same, so:

$$\begin{aligned}&\mathbb{E}_{X\sim p_0(X)}\mathbb{E}_{Y\sim p_0(Y|X)}[Y1[f(X)=\alpha]]=\mathbb{E}_{X\sim p_f(X)}\mathbb{E}_{Y\sim p_f(Y|X)}[Y1[f(X)=\alpha]]\\ \iff&\mathbb{E}_{X\sim p_0(X)}1[f(X)=\alpha]\,\mathbb{E}_{Y\sim p_0(Y|X)}[Y]=\mathbb{E}_{X\sim p_f(X)}1[f(X)=\alpha]\,\mathbb{E}_{Y\sim p_f(Y|X)}[Y]\\ \iff&\mathbb{E}_{X\sim p_0(X)}1[f(X)=\alpha]\,\mathbb{E}_{Y_0,Y_1|X}[(1-\pi_0(X))Y_0+\pi_0(X)Y_1]\\ &=\mathbb{E}_{X\sim p_f(X)}1[f(X)=\alpha]\,\mathbb{E}_{Y_0,Y_1|X}[(1-\pi_f(X)Y_0+\pi_f(X)Y_1]\end{aligned}$$

Since by assumption $p_0(X)=p_f(X)=p(X)$ we have that

$$\begin{aligned}\iff&\mathbb{E}_{X\sim p(X)}1[f(X)=\alpha]\,\mathbb{E}_{Y_0,Y_1|X}[(1-\pi_0(X))Y_0+\pi_0(X)Y_1]\\ &=\mathbb{E}_{X\sim p(X)}1[f(X)=\alpha]\,\mathbb{E}_{Y_0,Y_1|X}[(1-\pi_f(X))Y_0+\pi_f(X)Y_1]\\ \iff&\mathbb{E}_{X,Y_0,Y_1}1[f(X)=\alpha]((1-\pi_0(X))Y_0+\pi_0(X)Y_1)\\ &=\mathbb{E}_{X,Y_0,Y_1}1[f(X)=\alpha]((1-\pi_f(X))Y_0+\pi_f(X)Y_1)\\ \iff&\mathbb{E}_X 1[\mu_0(X)=\alpha]\mu_0(X)=\mathbb{E}_X 1[\mu_0(X)=\alpha]\mu_f(X)\end{aligned}$$

Where in the last line we substituted the definition of μ and used the assumption that $f(X)=\mu_0(X)$. Finally we note that by assumption $\pi_f(X)$ is non-constant. As X is binary it must be that f is injective. This implies that the expectation in the last line is given by the value of μ on a single point corresponding with α which proves that $\mu_0(X)=\mu_f(X)$.

Looking at the difference between $\mu_0(X)$ and $\mu_f(X)$ we see that:

$$\begin{aligned}&\mu_f(X)-\mu_0(X)=\\ &\quad((1-\pi_f(X))p(Y_0=1|X)+\pi_f(X)p(Y_1=1|X))-\\ &\quad((1-\pi_0(X))p(Y_0=1|X)+\pi_0(X)p(Y_1=1|X))\\ &=(\pi_f(X)-\pi_0(X))\,(p(Y_1=1|X)-p(Y_0=1|X))\end{aligned}$$

Hence the difference $\mu_f(X) - \mu_0(X)$ is zero iff at least one of the last two terms is zero. This means that f is calibrated on the deployment distribution iff for every x either $\pi_f(x) = \pi_0(x)$ or $p(Y_1 = 1|X = x) = p(Y_0 = 1|X = x)$

■

Supplemental Note S3 Numerical experiment

Experimental setup

We parameterize the joint distribution with a marginal distribution of X, a conditional of $T|X$ and $Y|T,X$, where we note that by assumption in the historic distribution, the treatment policy is *independent* of X, and also that the marginal distribution of X does not change after model deployment. Let $B(.)$ denote the bernoulli distribution and $\sigma(x) = \frac{1}{1+e^{-x}}$ the sigmoid (logistic) function.

$$x \sim B(p_x) \tag{72}$$
$$t = p_0(T) \in \{0, 1\} \tag{73}$$
$$\eta = \beta_0 + \beta_x x + \beta_t t + \beta_{xt} xt \tag{74}$$
$$y \sim B(\sigma(\eta)) \tag{75}$$

The parameter grid is:

parameter	distribution	interpretation	values
$p(X=1)$	$p(X)$	marginal distribution of X	$0.2, 0.5$
$p_0(T=1)$	$p_0(T)$	historic treatment policy	$0, 1$
β_0	$p(Y\|T,X)$	intercept on log odds scale	-0.5
β_x	$p(Y\|T,X)$	log odds ratio for X	$\log(1.1, 1.45, 1.8, 2.15, 2.5)$
β_t	$p(Y\|T,X)$	log odds ratio for T	log(1/2.5, 1/2.15, 1/1.8, 1/1.45, 1/1.1, 1, 1.1, 1.45, 1.8, 2.15, 2.5)
β_{xt}	$p(Y\|T,X)$	log odds for interaction between T and X	log(1/2.5, 1/2.15, 1/1.8, 1/1.45, 1/1.1, 1, 1.1, 1.45, 1.8, 2.15, 2.5)
higher Y		is higher Y better or worse	better, worse

Table S1: Parameters for numerical experiments

For these parameter values we first calculate the joint probability $p(X,Y)$ under the historic distribution. For some parameter values $p(Y=1|X=0) = p(Y=1|X=1)$, these settings are removed as they imply that the outcome risk is independent of X in the historical setting and would lead to a constant OPM-based treatment policy. When then also calculate outcomes under the new treatment policy with the outcome prediction model.

For each setting we calculate discrimination statistics (sensitivity, specificity, AUC), using:

$$\text{sens} = p(f(X) > \lambda|Y=1) \tag{76}$$
$$\text{spec} = p(f(X) < \lambda|Y=0) \tag{77}$$
$$\text{AUC} = \frac{1}{2}(\text{sens} + \text{spec}) \tag{78}$$

See Supplemental Note S2 for the derivation of the formula for AUC. First we need to determine whether the OPM-derived policy $\pi_f(X) = X$ (when $E[Y|X=1] > E[Y|X=0]$) or $\pi_f(X) = 1 - X$.

Then we compare the AUC before and after deployment to see if f is self-fulfilling, and the expected value of Y before and after deployment to see whether the new policy was harmful. Note that in our case harm for a subgroup is equivalent to marginal harm because the outcomes change for only one value of X (see Remark 9).

Results

.1 Experiment checks: Proposition 5 and Table 1

With these in hand, we can check Proposition 5 that: i) if the treatment effect is always positive, then (f, λ) is self-fulfilling; ii) if the treatment effect is always negative, then (f, λ) is not self-fulfilling.

sign(β_t)	sign($\beta_t + \beta_{xt}$)	not self-fulfilling (N)	self-fulfilling (N)
-1	-1	1508	0
-1	0	100	100
-1	1	200	200
0	-1	140	40
0	0	0	40
0	1	0	200
1	-1	320	44
1	0	0	180
1	1	0	1560

Table S2: Results of numerical experiments

We can also see that the numerical experiments follow Table 1.

higher Y is	$p_0(T = 1)$	selffulfilling	harmful (%)
worse	0	true	1
worse	0	false	0
worse	1	true	0
worse	1	false	1
better	0	true	0
better	0	false	1
better	1	true	1
better	1	false	0

Table S3: Results of numerical experiments, 2

.2 Plots

We now visualize the results. We include the following plots:

- Figure S2: A scatter plot of AUC on the historic data versus change in AUC in the post-deployment setting
- Figure S3: A scatter plot of the odds-ratio for treatment e^{β_t} for the group with $X = 0$, versus difference in AUC between pre and post deployment, with points colored by the treatment effect interaction term $e^{\beta_{xt}}$, and regions indicating whether the OPM-derived policy is harmful or not
- Figure S4: Like Figure S3 but with $e^{\beta_{xt}}$ on the x-axis and e^{β_t} as color codes.

Note that these three Figures include settings where the treatment is *always detrimental* (e.g. $\beta_t > 0, \beta_{xt} \geq 0$ and higher Y is worse). This means that no one should ever be treated with this treatment, making it highly unlikely that these treatments are in current clinical use so these settings are not very realistic. Instead, we subset the settings to those where the treatment is beneficial *on average*, although it does not have to be effective for both $X = 0$ and $X = 1$. This is typically the level of evidence available from RCTs before treatments are allowed on the market. These Figures are:

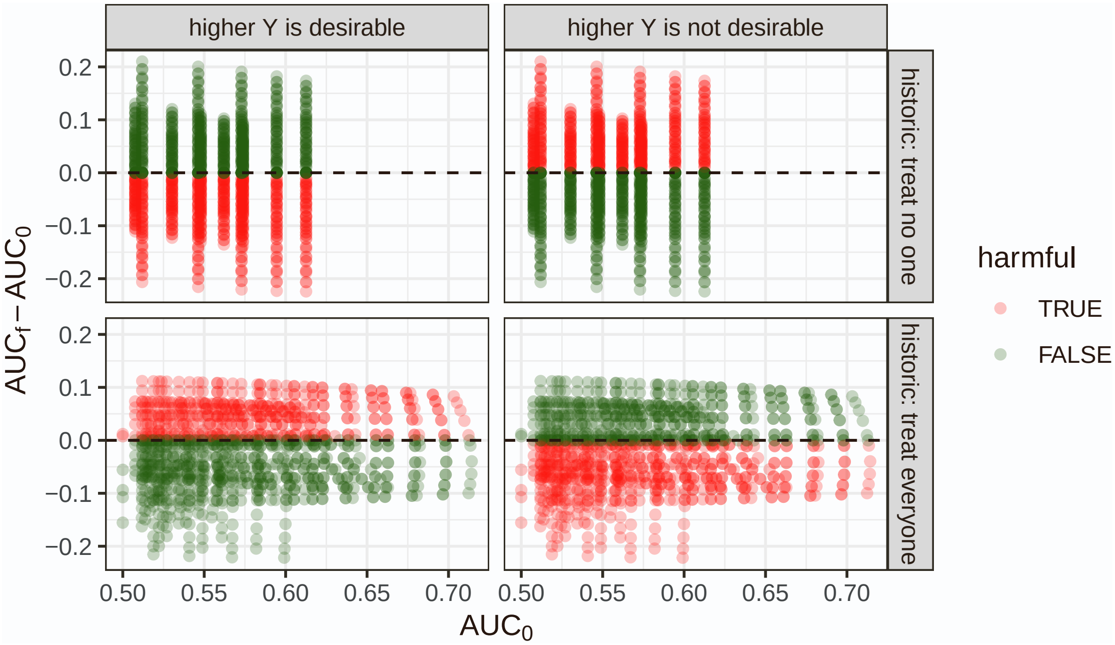

Figure S2: AUC under historic policy versus AUC increase under the policy with the outcome prediction model

- Figure S5: Like Figure S3 but subsetted to settings where treatment is are beneficial on average.
- Figure S6: Like Figure S4 but subsetted to settings where treatment is are beneficial on average.

Figure S5 is also presented in the main text.

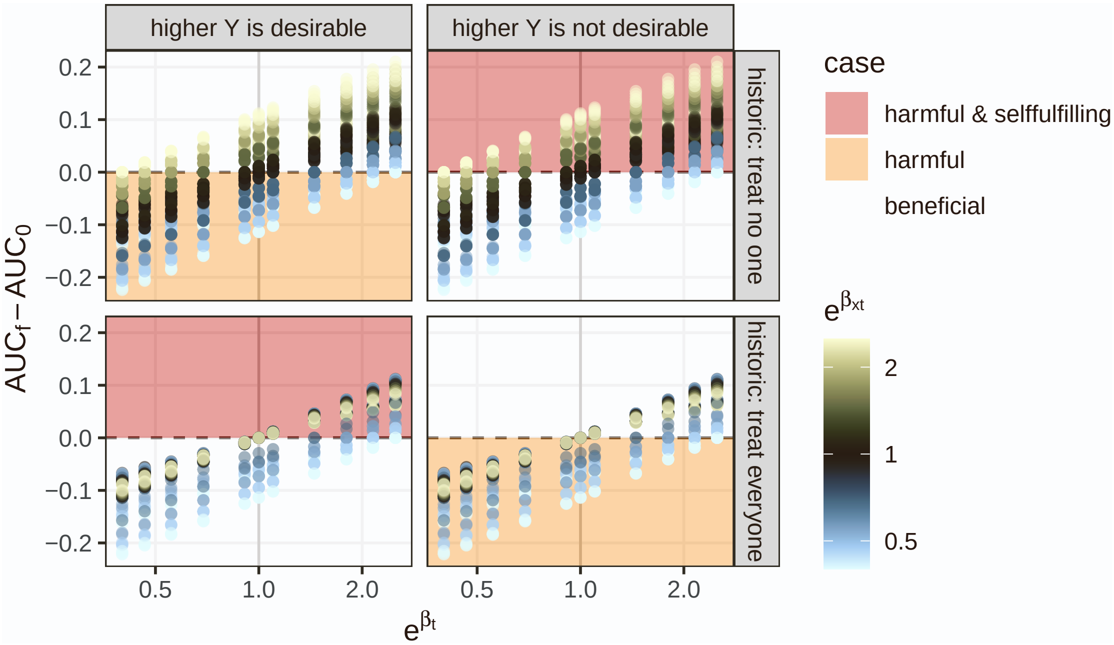

Figure S3: AUC difference versus treatment effect

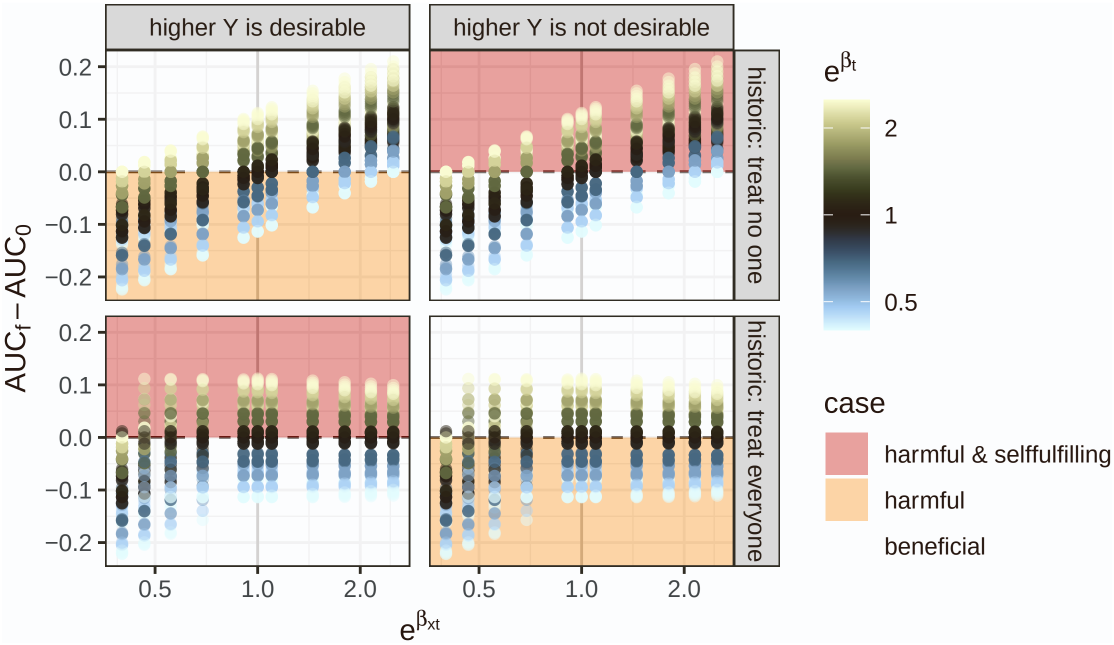

Figure S4: AUC difference versus treatment effect interaction

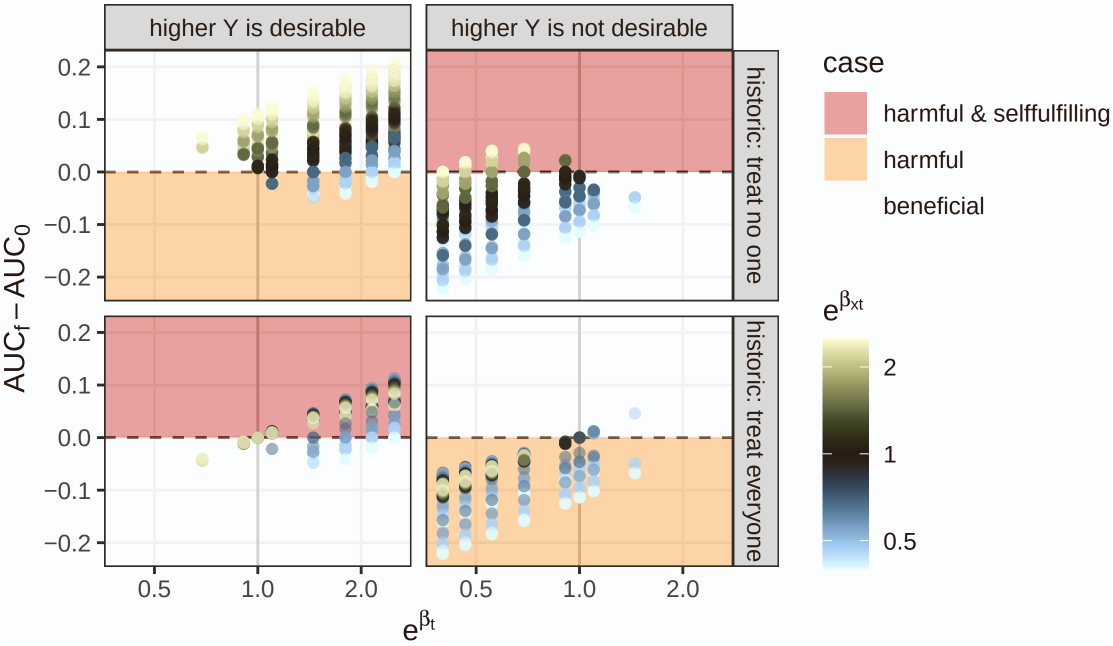

Figure S5: AUC difference versus treatment effect, only including settings where treatment is beneficial on average.

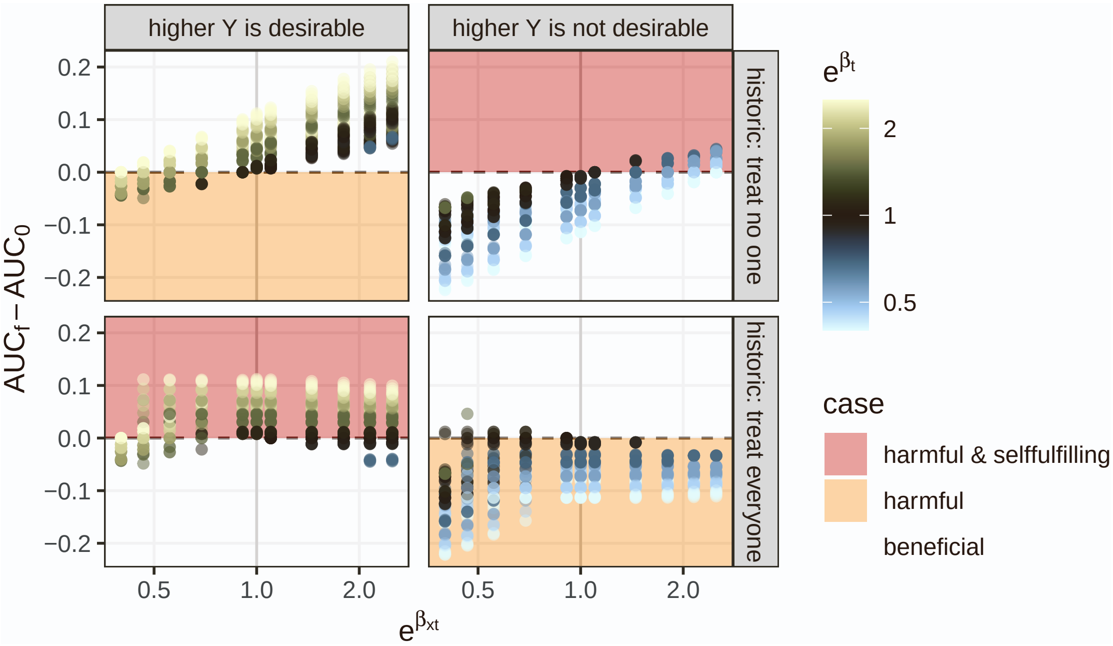

Figure S6: AUC difference versus treatment effect interaction, only including settings where treatment is beneficial on average.
